# Sorting of Carbon Nanotubes Based on Dispersant Binding Affinities

**DOI:** 10.1002/smsc.202400011

**Published:** 2024-03-08

**Authors:** Seongjoo Hwang, Seokhyeon Son, Minsuk Park, In‐Seung Choi, Sang‐Yong Ju

**Affiliations:** ^1^ Department of Chemistry Yonsei University 50 Yonsei‐ro Seodaemun‐Gu Seoul 03722 Republic of Korea

**Keywords:** binding affinities, carbon nanotubes, dispersants, dispersions, separations

## Abstract

Sorting single‐walled carbon nanotubes (SWNTs) that are heterogeneous into homogeneous groups based on their electronic type, chirality, and handedness is crucial for optoelectronic and biological applications. To achieve this, researchers have utilized dispersants with different binding affinities to sort SWNTs according to their chiralities. This review article provides an overview of the methods developed for sorting SWNTs using dispersants, with a particular focus on the role played by dispersant binding affinities. The article is organized into six sections, including an introduction, a background on SWNTs and dispersant‐based individualization of SWNTs, information on dispersants and their binding affinities, separation methods for SWNTs based on controlling the dispersant binding affinity, parameters used to control dispersant binding affinity, and a conclusion. It is hoped that this review will enhance understanding of the intricate interactions between dispersants and SWNTs, leading to the development of new SWNT sorting methods for future applications.

## Graphical abstract

It is essential to categorize diverse single‐walled carbon nanotubes (SWNTs) into uniform groups according to their electronic characteristics, chirality, and handedness. This review offers a comprehensive examination of techniques created for the segregation of SWNTs, emphasizing the significance of dispersant binding affinities in this process, particularly in relation to optoelectronic and biological applications.

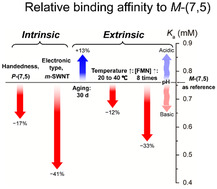

## 1 Introduction

Single‐walled carbon nanotubes (SWNTs) are one of several carbon allotropes that also include graphite, fullerene, and diamond. The generation of SWNTs in a carbon arc chamber was first discovered by S. Iijima of NEC in 1991 using transmission electron microscopy (TEM)^[^ ^1^ ^]^ after finding multiwalled carbon nanotubes (MWNTs).^[^ ^2^ ^]^ SWNTs are composed of cylindrical, rolled, *π*‐conjugated, hexagonal honeycomb carbon lattice structures in which C*sp* ^2^–C*sp* ^2^ bonds have distances of ≈1.42 Å. The seamless graphene array gives this material unique physical properties such as high electrical conductivity, mechanical strength, chemical inertness, and flexibility, along with interesting optical properties. Moreover, due to their nanometer diameters (*d*_t_), SWNTs have aspect ratios that exceed 100, which embodies them with narrow and long conducting channels.

Individualization of SWNTs according to electronic type, chirality, and handedness has been a goal of research efforts carried out in the last two decades. Full exploitation of this one‐dimensional (1D) nanomaterial hinges on the ability to sort SWNTs into groups composed of members having the same properties. For example, individualized semiconducting SWNTs with purities greater than 99.9999% are required for use as thin‐film transistors with channel widths below 10 nm.^[^ ^3^, ^4^, ^5^ ^]^ Typically, a prepared batch of SWNTs comprises 33.3% metallic (*m*)‐ and 66.7% semiconducting (*s*)‐tubes which are distinguished on the basis electronic properties. *m*‐ and *s*‐SWNTs have different optical properties and bandgaps according to the roll‐up mode of graphene, which is referred to as chirality in carbon nanotube community. Also, SWNTs are chiral and comprise mirror‐image pairs that have opposite roll‐up directions, termed handedness. Although methods to grow SWNTs that have uniform optoelectronic properties have been developed,^[^ ^6^ ^]^ their use in massive production is limited. A vast majority of studies in this area have concentrated on methods to sort homogeneous SWNTs utilizing dispersion with dispersants. In addition to utilizing single dispersants for this purpose, effective sorting methods that employ mixed‐dispersant systems have been devised to enhance sorting according to electronic type, chirality, and handedness.

In this review, methods for sorting SWNTs that are based on binding affinities of dispersant will be discussed and analyzed. First, chirality of SWNT, which is a key parameter governing physical properties, is explained in the context of SWNT heterogeneity and related optoelectronic properties and applications. The second section introduces a variety of relevant dispersants and describes relationships between their binding affinities and thermodynamic change associated with wrapping SWNTs. In addition, the major spectroscopic tools used for assessing binding affinities are compared and discussed in terms of signal cross sections. In the third section, various SWNT sorting methods are described in the context of their SWNT heterogeneity. Importantly, the intrinsic and extrinsic parameters controlling binding affinities and their scaling are discussed in the fourth section. Finally, a perspective is given for future efforts that focus on dispersant binding affinities and SWNT sorting. The discussion and analysis of binding affinities and their control by intrinsic and extrinsic parameters contained in this account should provide a deeper understanding of current schemes employed for sorting SWNTs according to electronic type, chirality, and handedness, which is expected to aid the design novel SWNT sorting protocols.

## 2 Background of SWNTs and Dispersant‐Based SWNT Individualization

### 2.1 SWNTs and Their Chirality

**Figure 1:**
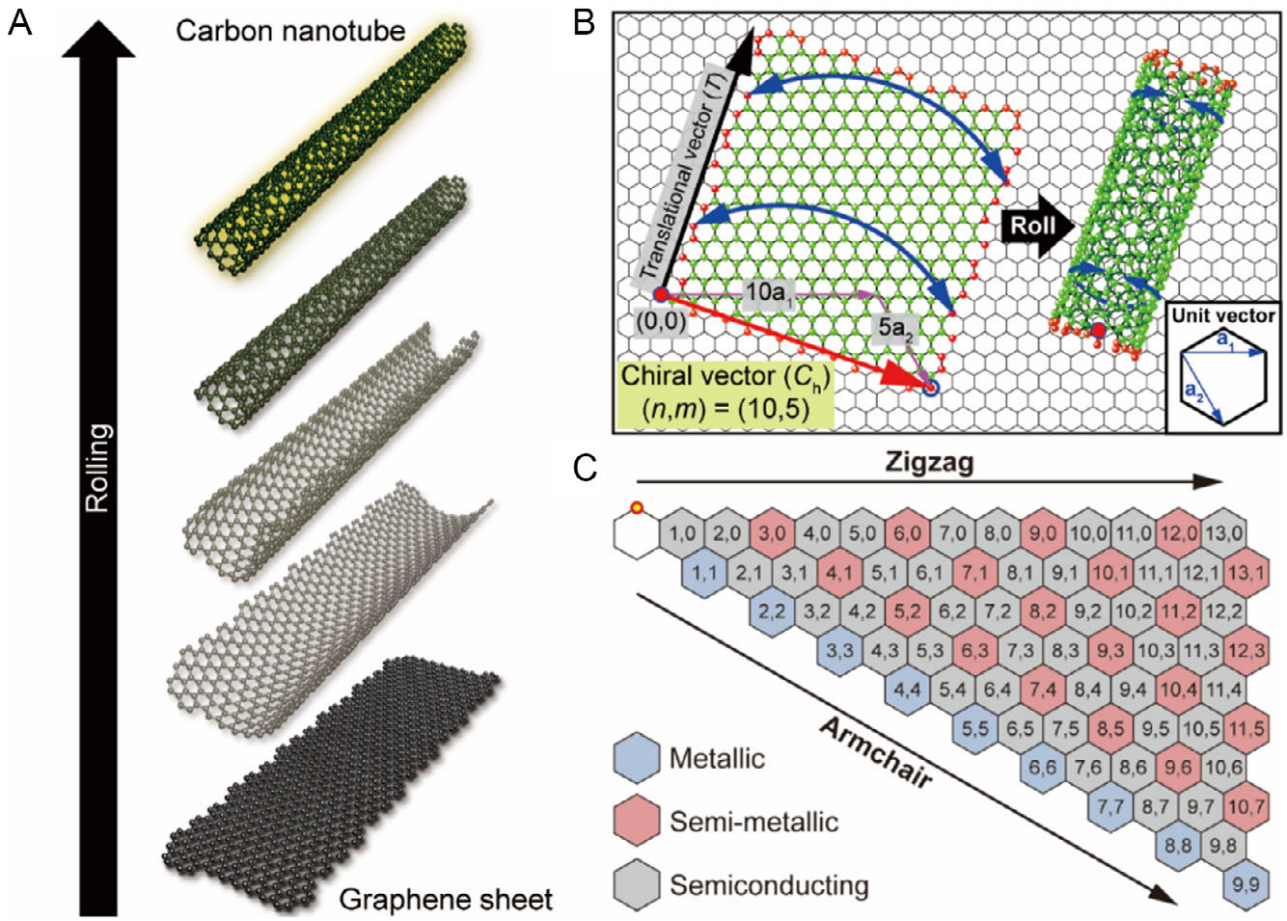
Formation of an SWNT along its chiral vector **C**_h_. A) Schematic of rolling a graphene sheet into an SWNT. B) Structure of SWNT rolled up along **C**_h_ on a graphene sheet. Inset: unit vectors **a**_1_ and **a**_2_ in a hexagonal graphitic structure. C) Various SWNT chiralities and their chirality‐dependent electronic types given in the graphene sheet.

SWNTs consist of cylindrically rolled‐up graphene sheets (**Figure** **1**A). Unlike other CNTs such as double‐walled carbon nanotubes (DWNTs) and MWNTs, SWNTs have a unique rolling direction defined by the chiral vector **C**_h_.^[^ ^7^, ^8^, ^9^ ^]^

(1) $$C_{h}=na_{1}+ma_{2}$$

where *n* and *m* are integers and **a**_1_ and **a**_2_ are unit vectors of the graphene unit cell (inset of Figure 1B). The subscript h in **C**_h_ (often referred to Hamada vector) corresponds to N. Hamada, who devised the *n*‐ and *m*‐terms for SWNTs having specific roll‐up directions.^[^ ^8^ ^]^ In general, *n* is greater than or equal to *m*. For instance, (10,5) SWNT is the resultant vector of two‐unit vectors (i.e., 10**a**_1_ and 5**a**_2_, magenta arrow in Figure 1B). Rolling‐up of a 2D graphene sheet in the direction of **C**_h_ and overlapping the starting and ending carbon atoms (red arrow in Figure 1B) produce the SWNT. By combining the various *n* and *m* values, numerous types of chiralities can be generated in a 30° sector fashion (Figure 1C). Using the (*n*, *m)* value, the *d*_t_ of the SWNT and the chiral angle (*θ*) of graphene sheet can be determined using the following equations.^[^ ^7^ ^]^

(2) $$d_{t}=\frac{\sqrt{3}a_{C-C}}{\pi }\sqrt{\left(m^{2}+mn+n^{2}\right)}$$

(3) $$\theta ={\text{tan}}^{-1}\left[\sqrt{3}m/\left(2n+m\right)\right]$$

where **a**_C—C_ is the bond length between two carbon atoms in the graphitic structure (i.e., 1.42 Å). Using, the (*n*, *m*) notation of **C**_h_, (*n*, *n*) denotes armchair SWNTs, (*n*, 0) denotes zigzag SWNTs, and all other (*n*, *m*) correspond to chiral SWNTs.

In addition, the translational vector **T**, which defines the longitudinal length of an SWNT unit cell, is calculated using the following equation.^[^ ^10^ ^]^

(4) $$T=\frac{\sqrt{3}C_{h}}{d_{R}}=\frac{\sqrt{3}\pi d_{t}}{d_{R}}$$

where *d*_R_ is the greatest common divisor (gcd) of (2*n* + *m*) and (2*m* + *n*) for a (*n*, *m*) SWNT. It is noteworthy that if there is gcd, one would have smaller **T** in SWNT unit cell. This is the reason why the initial literature on theoretical calculation utilized metallic (*m*)‐SWNT.^[^ ^11^ ^]^ In addition, one can visualize SWNT for a given (*n*, *m*) in the website.^[^ ^12^ ^]^

### 2.2 Diameter‐Dependent Density of States and Van Hove Singularity of SWNTs

**Figure 2:**
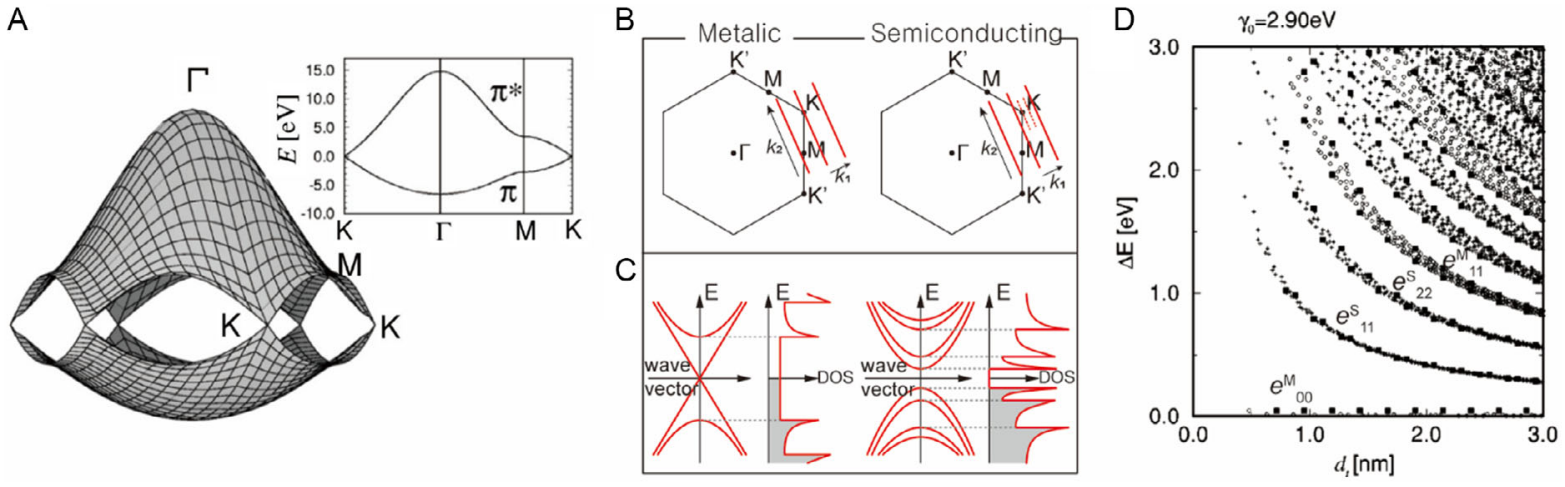
Optoelectronic properties of SWNTs governed by van Hove singularities (vHs). A) The energy dispersion relationship for 2D graphite based on *γ*_0_ = 3.013 eV, *s* = 0.129, *ε*_2p_ = 0 is given throughout the entire Brillouin zone. The inset shows energy dispersion along the high‐symmetry (i.e., Γ, M, and K) points. B) Wavevector‐dependent 2D Brillouin zone of *m*‐ and *s*‐SWNT. Note that wavevector **k**_2_ passes through the K point in case of *m*‐SWNTs whereas wavevector **k**_2_ passes the K point for *s*‐SWNTs. C) The corresponding DOS of *m*‐ and *s*‐SWNT. Spike‐like DOS is referred to as vHs. D) Kataura plot of *e*_*ii*_ versus *d*_t_ of a SWNT. Each (*n*, *m*) has a distinct set of *e*_*ii*_. Reproduced with permission.^[^ ^13^ ^]^ Copyright 2000, American Physical Society.

The optoelectronic structures of SWNTs can be approximated using calculations based on tight binding theory. The 1D density of states (DOS) of SWNTs are obtained using the zone folding method of the 2D energy dispersion relation of graphite. The energy dispersion relationship of graphite ($E_{g2D}^{\pm }\left(k\right)$) is calculated utilizing the eigenvalue equation (*H* − ES = 0) for the 2 × 2 Hamiltonian matrix *H*, eigenvalue *E*, and the 2 × 2 overlap integral matrix *S* between the wave vectors **k** and two inequivalent carbon atoms contained in the unit cell of graphite^[^ ^13^ ^]^ as follows.

(5) $$E_{g2D}^{\pm }\left(k\right)=\frac{\varepsilon_{2p}\pm \gamma_{0}\omega \left(k\right)}{1\mp s\omega \left(k\right)}$$

where $E_{g2D}^{+}$ and $E_{g2D}^{-}$ correspond to the respective valence (VB) and conduction (CB) bands in the π–π* transition of graphite, *ε*_2p_ is the site energy of the 2*p* atomic orbital, *γ*_0_ is the C–C transfer energy, and *s* is the overlap integral of the electronic wave function with the carbon atoms closest to each other. The probability density of the wave function *ω*(**k**) is determined by using the following equation

(6) $$\omega \left(k\right)=\sqrt{{\left|f\left(k\right)\right|}^{2}}=\sqrt{1+4\cos\frac{\sqrt{3}k_{2}a_{C[chemistry single bond solid line]C}}{2}\cos\frac{k_{1}a_{C[chemistry single bond solid line]C}}{2}+4\cos^{2}\frac{k_{1}a_{C[chemistry single bond solid line]C}}{2}}$$

where **k**_1_ and **k**_2_ are wave vectors along transverse and longitudinal directions of the SWNT. Inserting Equation (6) into the appropriate terms in Equation (5) results in the VB and the CB energies as a function of **k**_1_ and **k**_2_. In this way, the electronic energy dispersion relation for 2D graphite can be expressed by the wave vectors **k**_1_ and **k**_2_ in the hexagonal Brillouin zone (**Figure** **2**A). This map is the energy contour line of *E* ^+^ and *E* ^−^ values along Γ, M, and K, which are high‐symmetry points in the hexagonal Brillouin zone of the graphene. The Γ point has the largest π–π* transition and the K point has no bandgap because the VB and CB are in contact.

In SWNTs, the positions of arrays of **k**_1_ and **k**_2_ are determined by the quantum confinement effect imposed by *d*_t_^[^ ^14^ ^]^ (Figure 2B). When the **k**_2_ vector intersects the center of the K point, the electronic structure corresponds to *m*‐SWNT, when **k**_2_ vector passes the K point, the electronic structure corresponds to a semiconducting (*s*)‐SWNT. The 1D DOS, *D*(*E*), can be calculated in the unit of states/C‐atom/eV using the equation

(7) $$D\left(E\right)=\frac{T}{2\pi N}\sum_{\pm }\sum_{\mu =1}^{N}\int \frac{1}{\left|\frac{dE_{\mu }^{\pm }\left(k\right)}{dk}\right|}\delta \left(E_{\mu }^{\pm }\left(k\right)-E\right)dE$$

where **T** is a translational vector, *N* is the number of hexagons in the SWNT unit cell, and $E_{\mu }^{\pm }\left(k\right)$ is 1D energy dispersion relationship of a SWNT with *μ* = 1, 2, 3,…, *N*. The integrated value of *D*(*E*) over the energy region of *E*_*μ*_(**k**) is 2 for any (*n*, *m*) SWNT, which includes plus and minus *E*_g2D_ and spin degeneracy. The energy dispersion relationship per the wave vector **k** (i.e., $1/\frac{dE}{dk}$) is expressed in the denominator in the right side of Equation (7). van Hove singularities (vHs, see Figure 2C), where the DOS becomes singular when the differential value of *E*_*μ*_(**k**) over wave vector is zero, are observed, and the energy transition of SWNT occurs between the vHs. In addition, as several **k**_2_ vectors migrate away from the K point, the energy difference between the *π* and π* bands becomes larger, giving rise to vHs with different energies.

The optical transitions of SWNT can be expressed as a transition in the form of *e*_*ii*_ for vHs existing in VB and CB by putting order *i* (*i* is a positive integer) for the closest to the Fermi energy (*E*_F_). The transition from *i*‐th vHs in the VB to *i*‐th vHs in the CB occurs in accordance with the selection rules for the respective parallel (i.e., transition from *i*‐th of VB to *i*‐th of CB) and perpendicular (i.e., transition from the *i*‐th of VB to (*i* ± 1)th of CB) polarizations of light with respect to the nanotube axis.^[^ ^15^ ^]^ In general, considering that the transition is parallel to the longitudinal direction of the SWNT, the energy gaps between the highest vHs in the VB and lowest vHs in the CB in the cases of *m*‐SWNT and *s*‐SWNT (termed *e* ^M^_11_ and *e* ^S^_11_, respectively) are expressed in the following equations.

(8) $$e_{11}^{M}\left(d_{t}\right)=6a_{C-C}\gamma_{0}/d_{t}$$

(9) $$e_{11}^{S}\left(d_{t}\right)=2a_{C-C}\gamma_{0}/d_{t}$$

where *a*_C–C_ denotes C–C distance (i.e., 1.42 Å), *γ*_0_ denotes an overlap integral in eV, and *d*_t_ is a diameter of SWNT.

Optical transitions of *m*‐SWNT and *s*‐SWNT (Equation (8) and (9)) can be extended to higher‐order transitions.^[^ ^13^ ^]^ The relationship between *e* ^S^_*ii*_ and *e* ^M^_*ii*_ and the *d*_t_ of SWNT can be expressed as a Kataura plot (Figure 2D)^[^ ^16^ ^]^ in which each transition is expressed by different symbols. Optical transitions such as *e* ^M^_00_, *e* ^S^_11_, *e* ^S^_22_, *e* ^M^_11_, etc. are arranged in order of increasing energy with multiples of integers when they have the same *d*_t_. The exception is *e* ^M^_00_ which does not use integer pairs for the transitions originating from gap opening of *m*‐SWNT due to its curvature. Using this approach, the (*n*, *m*) index of SWNT can be utilized to analyze the transition energies corresponding to a specific *d*_t_ or vice versa.

Although the optoelectronic property calculations described above provide a framework for understanding SWNTs, they are based on the behavior of single electrons. However, many‐body interactions between electrons are dominant in SWNTs. In particular, bound electron–hole pairs called excitons profoundly affect the SWNT electronic structure. For instance, tight‐binding theory predicts multiples of integer relationships between *e* ^S^_11_ and *e* ^S^_22_ for a given SWNT *d*_t_. However, experimental results show that the ratio of *e* ^S^_11_ to *e* ^S^_22_ is ≈1.8 depending on the SWNT chirality,^[^ ^17^ ^]^ which was expressed as a “ratio problem”.^[^ ^18^ ^]^ Those evidence manifests the importance of excitonic nature of SWNT. Moreover, optoelectronic anisotropy exists near the K point, creating a trigonal warping effect,^[^ ^13^ ^]^ which influences variations of the *e* ^S^_11_/ *e* ^S^_22_ ratio according to SWNT (*n*, *m*). Nevertheless, (*n*, *m*) is an important index for defining SWNTs in terms of their optoelectronic properties.

### 2.3 Heterogeneity of SWNTs

The characteristics of SWNTs (i.e., electronic type, *d*_t_, and optical transitions) depend on the Hamada vector **C**_h_. In other words, SWNT mixtures containing different (*n*, *m*) species have nonuniform physical properties.^[^ ^19^ ^]^ Many efforts to control the intrinsic properties of SWNTs (i.e., absorption,^[^ ^20^, ^21^ ^]^ Raman scattering,^[^ ^22^, ^23^, ^24^, ^25^ ^]^ Rayleigh scattering,^[^ ^26^, ^27^ ^]^ and electronic transport^[^ ^28^, ^29^, ^30^ ^]^) have been conducted over the last three decades.

A variety of SWNTs with differences in chiralities arise in batch type processes employed for their synthesis. Growth of SWNTs is closely related to structural properties of metal catalysts used in their synthesis (i.e., element composition, catalyst size, crystallinity, etc.). In particular, to achieve chirality‐specific growth, not only does the uniform size and homogeneous composition of a catalyst need to be controlled,^[^ ^31^, ^32^ ^]^ but also the epitaxy between the crystal structure of the metal catalyst and the carbon nanotube chirality is an essential factor requiring consideration.^[^ ^6^ ^]^ Currently, the growth methods used to prepare SWNTs include arc discharge,^[^ ^2^, ^33^ ^]^ laser ablation,^[^ ^34^ ^]^ and chemical vapor deposition (CVD) methods.^[^ ^35^, ^36^ ^]^ As more efficient SWNT growth methods are developed and the mass production capability increases, the price of SWNTs will continue to decrease^[^ ^37^ ^]^ and studies and applications of these materials will be accelerated. Although growth methods using bimetallic alloy catalysts (such as Co–Mo^[^ ^38^ ^]^ or Co–W^[^ ^6^ ^]^) and templated end‐cap precursors^[^ ^39^ ^]^ have enabled generation of SWNTs having specific chiralities, its commercialization with large‐scale reproducibility will require more reseach. Nevertheless, because direct production of homogeneous as‐synthesized SWNTs for use in high‐end applications remains challenging, protocols for generating single‐species SWNTs that are based on sorting of as‐grown SWNTs are being actively pursued. Moreover, other byproducts/impurities are present in the as‐synthesized SWNTs batch including residual metal catalysts and carbonaceous materials.^[^ ^33^, ^40^ ^]^ Although these impurities can be removed by oxidation^[^ ^41^, ^42^, ^43^, ^44^, ^45^, ^46^ ^]^ and microfiltration^[^ ^47^, ^48^, ^49^ ^]^ methods to that promote complete removal are need to produce high purity SWNTs.

**Figure 3:**
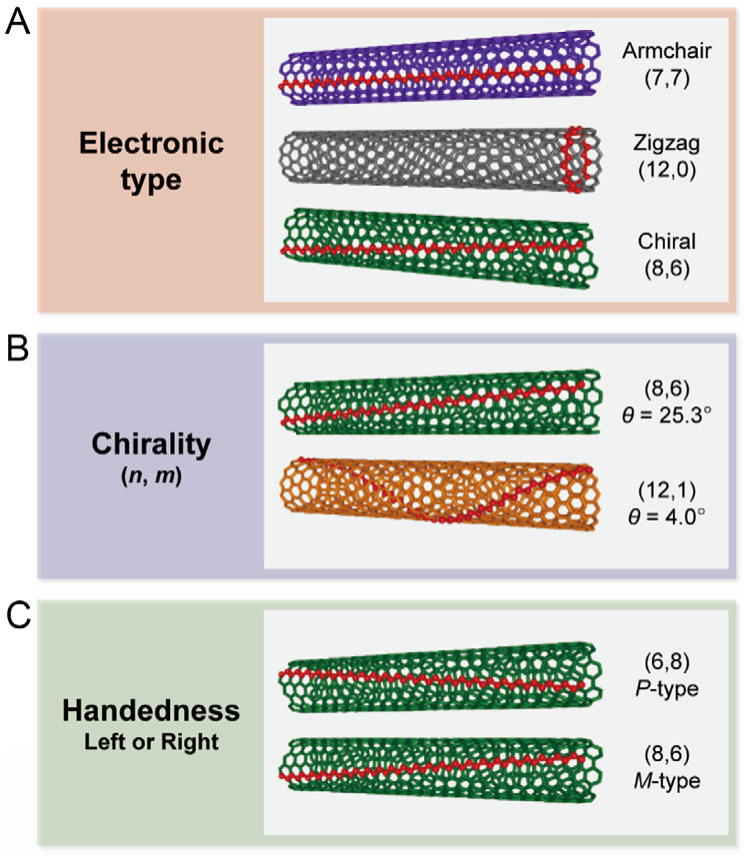
Classification of SWNTs according to (*n*, *m*)‐induced differences: A) electronic type, B) chirality, and C) handedness. The acetylene line is shown in red line for visual clarification of graphene roll‐up differences.

As shown in **Figure** **3**, SWNTs are categorized according to electronic type, chirality, and handedness. *s*‐ and *m*‐SWNT are present in a ratio of 2:1 unless chirality selection of SWNT occurs during growth. In the figure, color coding of SWNTs having similar *d*_t_ (≈1 nm) is utilized to distinguish different physical properties. SWNTs with different *θ* values are distinguishable by the orientation of the acetylenic line of the graphene sheet which relates to the roll‐up direction. Furthermore, differences in electronic type (Figure 3A) create a large inhomogeneity in the physical properties of SWNTs. In addition, in terms of chirality (*n*, *m*), when the difference between *n* and *m* is a multiple of 3 including 0 (i.e., (7,7) and (12,0) in Figure 3A), the SWNT has a metallic property without a bandgap. If not a multiple of 3, the SWNT is semiconducting (i.e., (8,6)).

The existence of various chiralities is also responsible for heterogeneity (Figure 3B). In the case of the optical properties, each SWNT chirality has a different transition energy between its vHs.^[^ ^13^ ^]^ For instance, although (8,6) and (12,1) chiralities have similar *d*_t_ (i.e., 0.95 nm and 0.98 nm), they exhibit different optical transitions (e.g., *e* ^S^_22_ of (8,6) and (12,1) SWNT is 713 and 799 nm, respectively^[^ ^24^ ^]^), which correspond to different responses in high‐end optoelectronic applications. There also is a source of heterogeneity called handedness, which corresponds to the enantiomers of chiral SWNTs (Figure 3C), which are nonsuperimposable mirror image forms that have opposite **C**_h_ and oppositely directed acetylenic lines (red in Figure 3C).^[^ ^50^, ^51^ ^]^ Unlike *R*‐ and *S*‐nomenclature used to classify enantiomers of chemical substances, supramolecule nomenclature is used to designate optical isomers of SWNTs^[^ ^52^, ^53^ ^]^ as being right‐handed or *P* (or +) and left‐handed *M* (or −) supramolecules.^[^ ^52^ ^]^ Without using supramolecular nomenclature, the handedness can be expressed as (*m*, *n*) for *P*‐ and (*n*, *m*) for *M*‐(*n*, *m*). The optical and electrical properties of SWNT enantiomers are equivalent except in their response to circularly polarized light, like in circular dichroism (CD) spectroscopy where the relative abundances of the enantiomers, referred to as enantiomeric excess (*ee*), determine the magnitude of the CD signal. Also, due to the relatively low signal‐to‐noise ratio of CD signals, characterization of SWNT optical isomers using this method requires a substantially high *ee* enrichment. For this reason, the generation of optical isomers of SWNT often requires sorting according to both chirality and handedness.

## 3 Dispersants, Binding Affinity, and Determination

### 3.1 Various Dispersants to Individualize SWNT

Owing to the large of numbers of applications, a great demand exists for methods that enable generation of highly homogeneous single SWNTs from heterogeneous as‐synthesized SWNTs. While having outstanding physical properties,^[^ ^54^ ^]^ atomically smooth *π*‐conjugated graphitic structures have a propensity to undergo aggregation‐induced bundling due to strong intertube van der Waals (vdW) interactions. The results of a computational study showed that bundled SWNTs have a strong binding energy of 950 meV per each nanometer between two (10,10) tubes.^[^ ^55^ ^]^ Bundling causes a deterioration of characteristic physical properties such as PL,^[^ ^56^ ^]^ tensile strength,^[^ ^57^ ^]^ etc.

Several strategies have been proposed to overcome SWNT bundling, one which involves side wall functionalization using oxidation methods.^[^ ^58^, ^59^, ^60^ ^]^ Although side wall oxidation makes SWNTs dispersible, C*sp* ^3^‐bonded functional groups act like defects that cause properties of the SWNTs to be diminished. Another yet most prevalent method utilizes noncovalent functionalization of SWNT side walls with dispersants.^[^ ^61^, ^62^, ^63^ ^]^ Dispersant‐based sorting/individualization is achieved using a simple sonication–ultracentrifugation protocol, which produces homogeneous SWNTs wrapped by dispersants.

**Figure 4:**
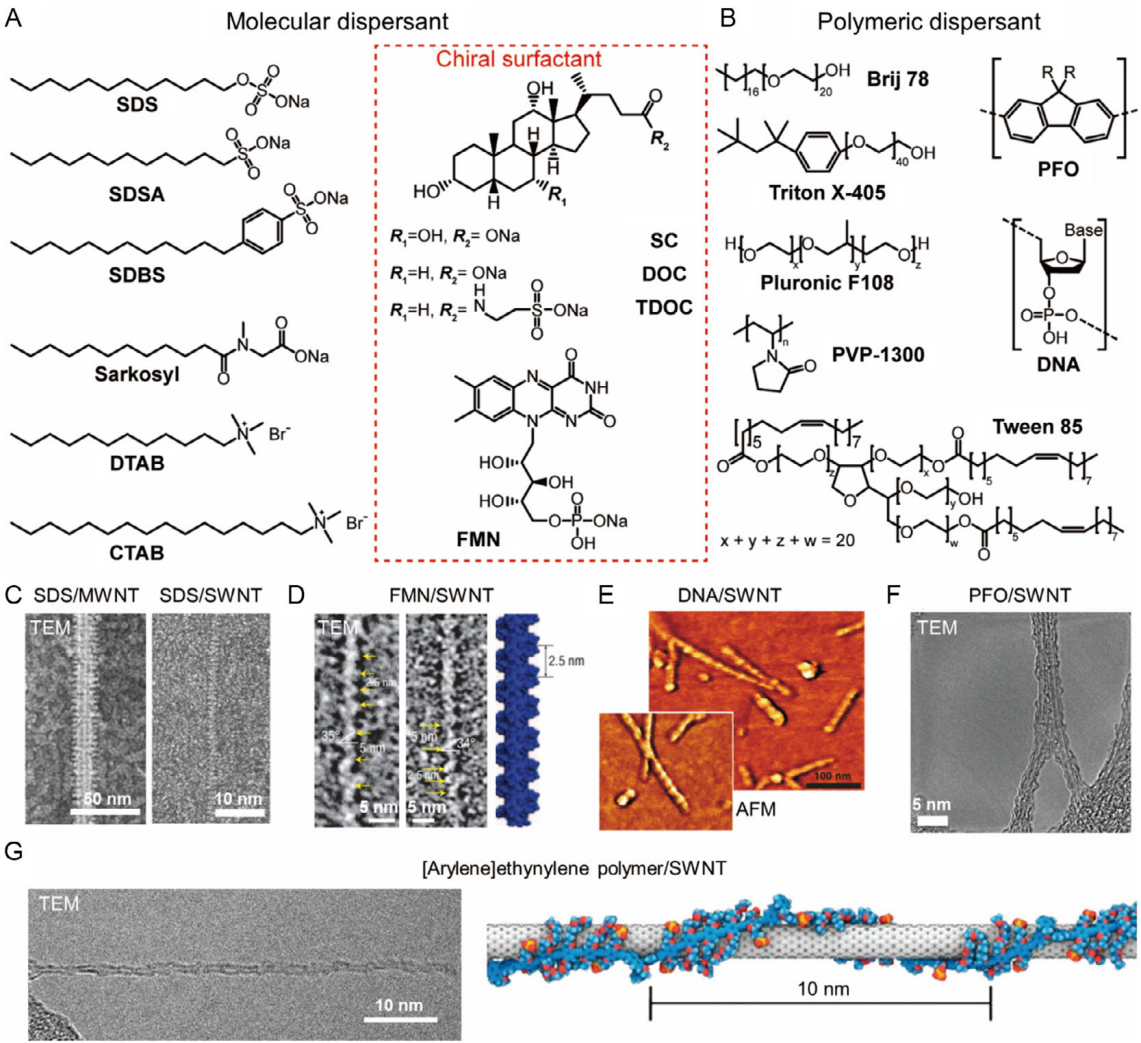
Various dispersants used for SWNT individualization. Molecular structures of representative A) molecular dispersants and B) polymeric dispersants commonly used for SWNT dispersion. Various dispersant organization on SWNT. C) TEM images of the supramolecular organization of SDSs on MWNT (left) and SWNT (right). Reproduced with permission.^[^ ^64^ ^]^ Copyright 2003, American Association for the Advancement of Science D) TEM images of uranyl acetate‐stained FMN‐wrapped SWNTs (left) and simulated FMN helical configurations (right). Reproduced with permission.^[^ ^65^ ^]^ Copyright 2008, Springer Nature. E) AFM phase image of SWNTs wrapped by DNA, having a regular helical pitch of *≈*18 nm and height of *≈*2 nm. Reproduced with permission.^[^ ^67^ ^]^ Copyright 2003, American Association for the Advancement of Science. F) TEM image of two PFO‐wrapped SWNTs in contact with each other. Reproduced with permission.^[^ ^69^ ^]^ Copyright 2012, Royal Society of Chemistry. G) TEM image of binaphthalene‐based chiral polymer wrapped (6,5) SWNTs. Reproduced with permission.^[^ ^70^ ^]^ Copyright 2018, American Chemical Society.

The dispersants commonly used for dispersing SWNTs can be classified ino two types including molecular dispersants (i.e., sodium dodecyl sulfate (SDS),^[^ ^20^, ^64^ ^]^ sodium dodecyl benzene sulfonate (SDBS),^[^ ^63^ ^]^ sodium cholate (SC), and flavin derivatives^[^ ^65^, ^66^ ^]^ (**Figure** **4**A), and polymeric dispersants (i.e., single‐stranded DNA (ssDNA),^[^ ^67^ ^]^ conjugated polymer,^[^ ^68^, ^69^ ^]^ and chiral polymer^[^ ^70^ ^]^) (Figure 4B). Most of the molecular dispersants used for this purpose are composed of hydrophobic long alkyl chains and ionic hydrophilic head groups such as phosphate, carboxylate, sulfate, and sulfonate, which form micelles in solution. The hydrophobic alkyl chains undergo vdW interactions with SWNT to form micellar structures on the SWNT surface. Richard and co‐workers^[^ ^64^ ^]^ used cryo‐technology and TEM to show that SDSs or lipid organizations exist as cylindrical micelles or hemimicelles on the side walls of SWNTs (Figure 4C).

Moreover, several molecular dispersants are chiral due to the presence of a stereogenic center in their structures. Representative chiral dispersants are SC, sodium deoxycholate (DOC), sodium taurodeoxycholate (TDOC), and flavin mononucleotide (FMN) (see red box in Figure 4A). These dispersants have hydrophobic rigid multiring structures that interact with SWNT and hydrophilic side chain groups to promote dispersal of the SWNTs in aqueous solution. Chiral flavin derivatives interact with SWNTs in a more distinctive way. Unlike other molecular dispersants (Figure 4A) which adsorb on SWNT surface by vdW interactions, the *π*‐conjugated isoalloxazine ring in the flavin is adsorbed on the SWNT surface through *π–π* interactions^[^ ^71^, ^72^, ^73^ ^]^ to form a unique helical structure. As inspection of the TEM image and simulated FMN helical configuration shown in Figure 4D reveals, periodic undulation patterns from flavin (yellow arrow) with a projected pitch angle (*≈*36°) are created.^[^ ^65^ ^]^

The next family of SWNT dispersal agents are polymeric dispersants that are composed of repeats of hydrophilic and hydrophobic moieties. In the beginning of efforts targeted at individualizing SWNT, ssDNA consisting of hydrophobic nucleotides and hydrophilic deoxyribityl phosphate backbone were employed as dispersants to disperse SWNTs. Zheng and co‐workers^[^ ^67^ ^]^ demonstrated that the structure of self‐assembled ssDNA‐SWNT is uniformly periodic with a regular pitch, and ssDNA adsorption on the SWNT surface is dependent on the DNA sequence (Figure 4E). In addition, several polymeric dispersants containing *π*‐conjugated ring systems have been utilized to disperse and sort SWNTs through advantageous of *π–π* interactions. For example, Koyama and co‐workers^[^ ^69^ ^]^ determined the structure of polyfluorene (PFO)‐wrapped SWNT structures using TEM. The TEM image (Figure 4F) shows that PFO is adsorbed tightly on the SWNT surface as a consequence of *π–π* interactions of three rings in PFO. More recently, Bai and co‐workers^[^ ^70^ ^]^ demonstrated using TEM that the binaphthalene‐based chiral polymer creates a packed wrapping on the SWNT surface (Figure 4G).

The observations made in these studies suggest that the structure of the dispersant in the complex is governed by the regular supramolecular structure of the SWNT. In other words, dispersants spontaneously incoporate into self‐assembled structures that have distinctive motifs. The formation of supramolecular self‐assembled dispersant‐SWNT structures through weak molecular forces (i.e., hydrogen bonding, vdW, dipole–dipole, etc.) is an equilibrium process. Therefore, several features can perturb this equilibrium and therefore govern the binding affinity and consequent SWNT sorting ability of the dispersant. As a result, a thorough understanding of the factors governing interactions occurring between dispersant and SWNT is required to assess binding affinities and to design the tailored dispersants and the dispersing methods.

### 3.2 Binding Affinity Between SWNTs and Dispersants for SWNT Sorting

Sorting methods using dispersants have received great attention because it can be utilized for large‐scale individualization of SWNTs. The appropriate choice of dispersant is an important component of sorting SWNT into specific groups. Although the relative dispersion efficiencies of dispersants for SWNTs have been investigated,^[^ ^63^, ^74^ ^]^ and the origins of sorting selectivity have not been fully resolved. Therefore, knowledge about the nature of supramolecular self‐assemblies of dispersants and the binding affinities of dispersants to SWNTs is essential to gain an understanding of underlying principles.

The relative binding affinity (*K*_a_) concept was initially used to isolate single SWNT types by codispersant titration of FMN‐containing dispersions.^[^ ^65^ ^]^ When a codispersant (e.g., SDBS) is added to the FMN–SWNT dispersion, FMN on the SWNT surface is replaced by the codispersant via equilibrium processes expressed as

(10) $$\text{FMN}+\text{SWNT}\overset{K_{a}}{⇌}\text{FMN}-\text{SWNT}$$

(11) $$\text{FMN}-\text{SWNT}+\text{codispersant}\overset{K_{\text{FMN}}-\text{cosurfactant}}{⇌}\text{codispersant}-\text{SWNT}+\text{FMN}$$

where *K*_FMN‐codispersant_ is equilibrium constant for dispersant replacement and *K*_a_ is [FMN]/*K*_FMN‐codispersant_.^[^ ^75^ ^]^ The process can be monitored using the PL of the SWNT, which undergoes a large redshift upon wrapping with FMN, and a subsequent intensity and wavelength change when an alkyl dispersant like SDBS become bound due to a change in the local dielectric constant. In this process, the change of PL intensity versus dispersant concentration follows a sigmoidal relationship that can be quantified using Hill analysis. The Hill equation is

(12) $$\theta ={\left[{\left(\frac{K_{a}}{\left[\text{titrant}\right]}\right)}^{r}+1\right]}^{-1}$$

where *K*_a_ is the midpoint of the sigmoidal transition, *θ* is PL fraction based on the PL intensity of FMN‐ and titrant‐SWNT, and *γ* is a Hill coefficient representing dispersant cooperativity. A larger *K*_a_ value corresponds to stronger binding affinity between FMN and SWNT. In other words, the binding affinity of codispersant with SWNT is expressed as *K*_d_, which has a reciprocal relationship to *K*_a_.

**Figure 5:**
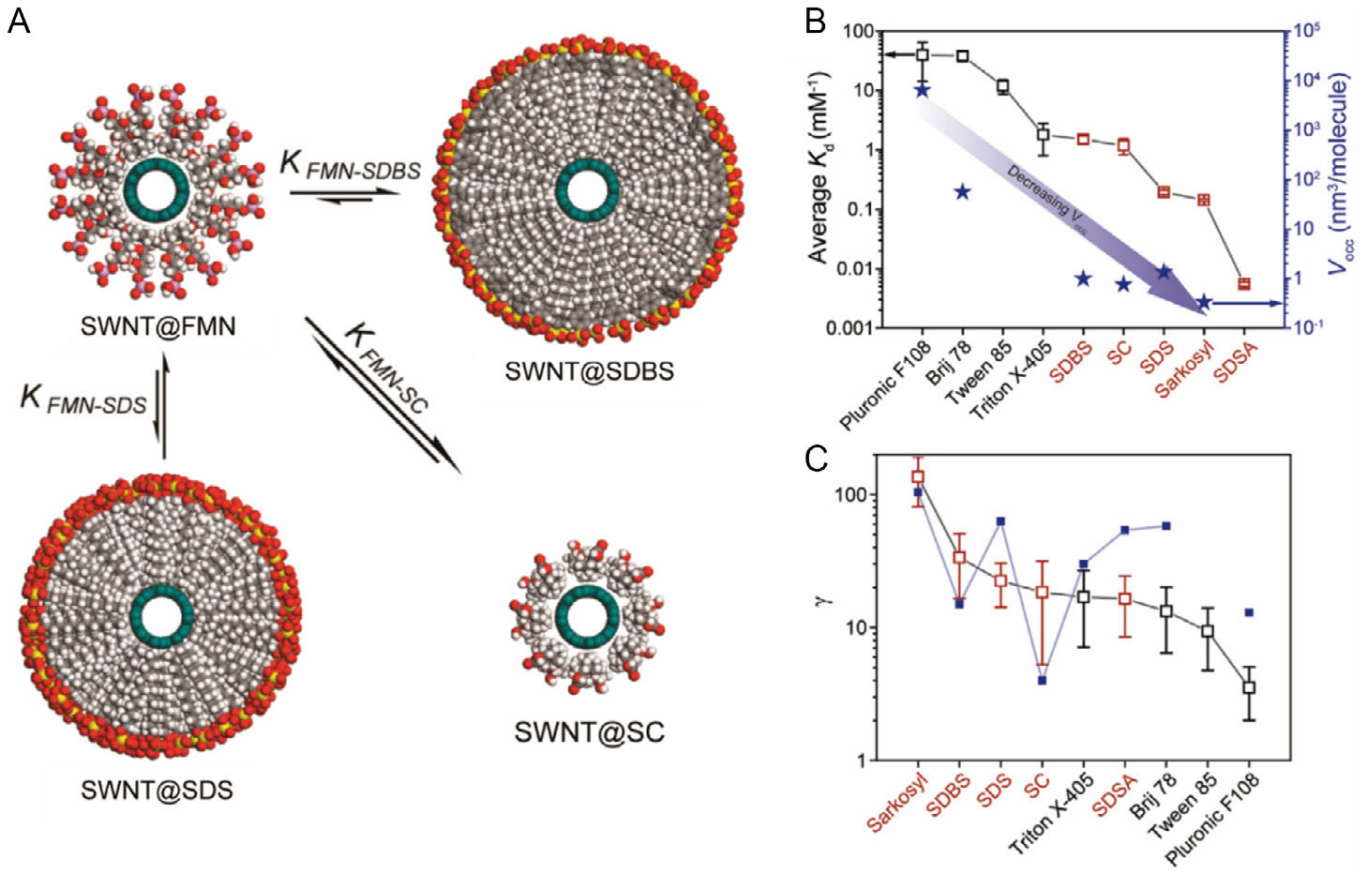
The *K*_a_ difference according to characteristics of the dispersion. A) Schematic of various dispersants surrounding the SWNT. Double arrows and their relative magnitudes indicate that the process is in equilibrium with an equilibrium constant of *K*_init‐final_ (init and final describe the initial and final dispersants, respectively) and the reaction direction. Reproduced with permission.^[^ ^75^ ^]^ Copyright 2013 American Chemical Society. B,C) Comparison of *K*_d_ and *γ* for various dispersants with SWNT. B) Average *K*_d_ (left axis) and *V*_occ_ (star symbol, right axis) according to dispersants. C) *γ* of the various nonionic (black) and anionic (red) dispersants in the presence (empty square) or absence (solid square) of SWNTs. Bars indicate standard deviations. Reproduced with permission.^[^ ^76^ ^]^ Copyright 2018, Elsevier.

*K*_a_ of FMN varies according to the SWNT and codispersant type.^[^ ^75^, ^76^ ^]^ In **Figure** **5**A, a schematic of dispersant organizations on the SWNT surface created during dispersant replacement is shown. The supramolecular structure and *K*_d_ depend on the nature of the dispersant. If a dispersant contains functional groups that preferentially interact with the SWNT surface (i.e., benzene ring of SDBS or steroid group in SC), the process will have a higher *K*_d_ value. In an investigation reported by Park and co‐workers,^[^ ^76^ ^]^ relative *K*_d_ values for equilibria involving various dispersants and SWNTs were determined and scaled using an optical titration method. In this study, dispersants with high molecular weights (black) were found to have larger *K*_d_ values and occupied volumes of dispersants (*V*_occ_) than those with lower molecular weights (red) (Figure 5B). The *γ*, which means that the aggregation number as dispersant cooperativity, for higher‐molecular‐weight dispersants, was lower than those of dispersants with lower molecular weights (Figure 5C). The results of this study suggest that structure and molecular weight changes of dispersants can lead to changes of *K*_d_ by four orders of magnitude. Thus, understanding the differences in *K*_a_ governed by competition between host dispersants and guest codispersants binding to various SWNTs leads to the principles needed to design systems used for sorting of SWNMs by type.

### 3.3 SWNT Analysis Using Various Spectroscopies

**Figure 6:**
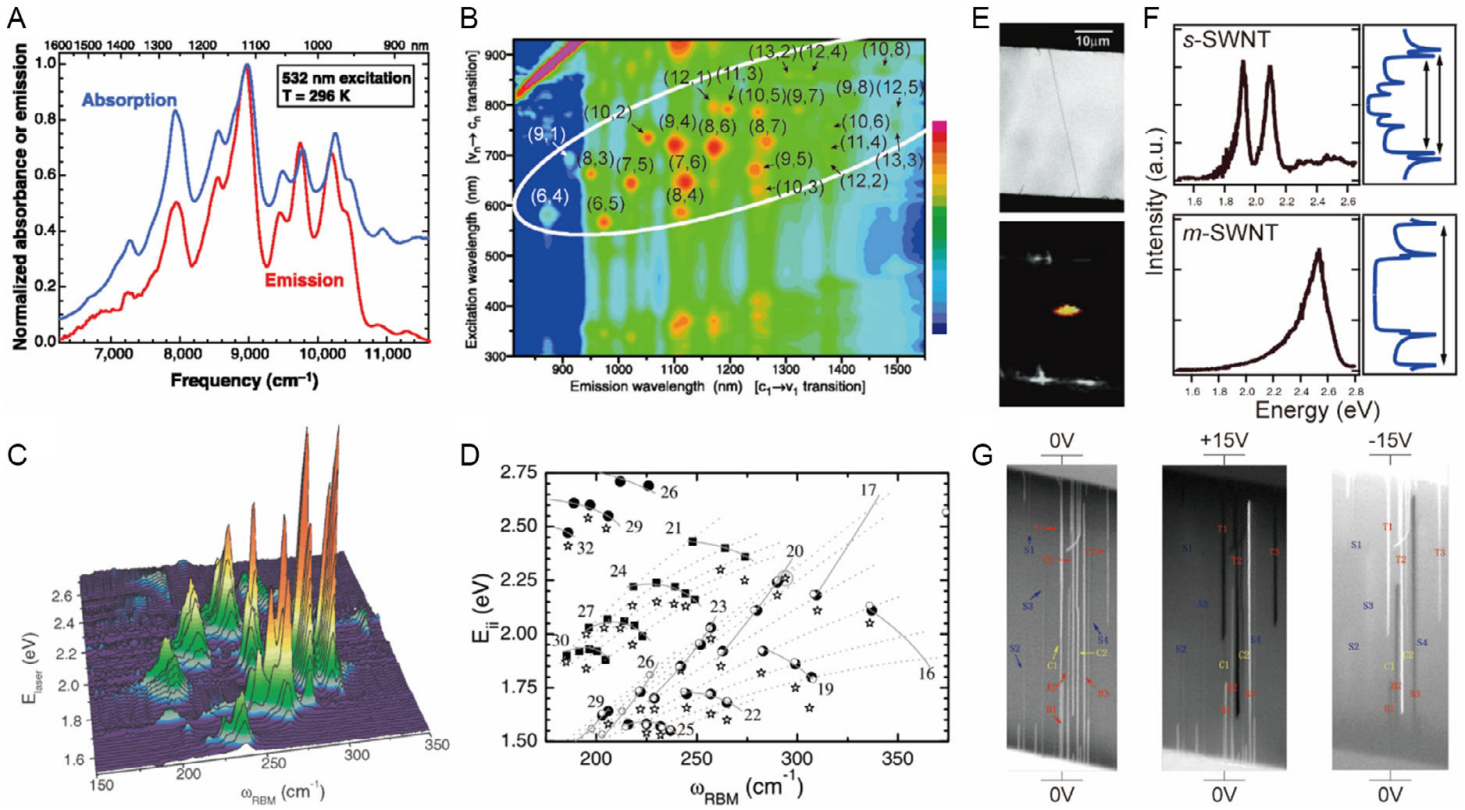
Spectroscopic methods for the analysis of the optical properties of SWNTs. A) Absorption spectrum (blue) and emission spectrum (red, excitation at 532 nm) of individual SWNTs suspended in SDS micelles in D_2_O. Reproduced with permission.^[^ ^20^ ^]^ Copyright 2002, American Association for the Advancement of Science. B) Contour plot of fluorescence intensities versus excitation and emission wavelengths for SWNTs dispersed by SDS in D_2_O. Reproduced with permission.^[^ ^61^ ^]^ Copyright 2002, American Association for the Advancement of Science. C) RBM measurements of SWNTs dispersed in SDS aqueous solution using Raman spectroscopy using 76 different laser lines whose energies are denoted by *E*_laser_. D) *e*_ii_ versus *ω*_RBM_ for 46 different (*n*, *m*) SWNTs measured using RRS. Reproduced with permission.^[^ ^23^ ^]^ Copyright 2004, American Physical Society. E) SEM (top) and Rayleigh scattering images (bottom) of individualized SWNTs suspended on a Si substrate. F) Rayleigh scattering spectra (left) and DOS diagrams (right) of *m*‐ and *s*‐SWNT. Reproduced with permission.^[^ ^82^ ^]^ Copyright 2004, American Association for the Advancement of Science. G) Determination of electronic type of SWNT using voltage contrast SEM measurements. Reproduced with permission.^[^ ^85^ ^]^ Copyright 2012, American Chemical Society.

As mentioned above, optical properties of individualized SWNT can be predicted using theoretical calculations and Kataura plots. However, SWNT bundling hampers experimental verification of the theoretically predicted properties but information about binding can be realized by taking advantage of noncovalent functionalization methods to generate individualized SWNT. O’Connell and coworkers^[^ ^20^ ^]^ pioneered the dispersant micelle‐based individualization method. Using this protocol, individualized SWNT were obtained using ultrasonic agitation of an aqueous mixture of as‐prepared SWNTs and SDS, followed by subsequent centrifugation to remove SWNT bundles and residual catalyst. The dispersions have distinct absorption and fluorescence bands that correspond to electronic transitions between vHs for individual (*n*, *m*) SWNTs (**Figure** **6**A).

In another effort, Bachilo and co‐workers^[^ ^61^ ^]^ utilized PL excitation (PLE) spectroscopy to assign (*n*, *m*) indices for multiple *s*‐SWNTs using fluorescence signals corresponding to their bandgap energies (Figure 6B). The results showed that SDS–SWNT dispersions exhibited distinct absorption and emission signals associated with electronic transitions from various *s*‐SWNT species. PLE contour maps revealed not only *e* ^S^_11_, *e* ^S^_22_, and *e* ^S^_33_ electronic transitions, but also that (*n*, *m*) relationships indicate family (Fam = 2* n* + *m*) and modality (mod = remainder of |*n − m*|) of the SWNTs. Later, the PL‐based method has been utilized to determine exciton binding energy^[^ ^77^ ^]^ and exciton diffusion length^[^ ^78^ ^]^ of SWNTs.

Another method to characterize (*n*, *m*) of SWNTs involves the use of resonance Raman spectroscopy (RRS), in which inelastic scattering is enhanced by matching excitation energies of a substance with those of optical transitions. The Raman spectrum of as‐prepared SWNTs, contains several prominent bands near 1350, 1590, and 2650 cm^−1^, with the latter arising from defect‐initiated bands or D band, G band (graphitic band commonly observed in graphitic materials), and G′ (or 2D for graphene community, overtone of the D and G bands). The positions of the D and G’ bands are dependent on the excitation energy as a consequence of the energy dependency of a two‐phonon process.^[^ ^79^ ^]^ While these bands are commonly observed in resonance Raman spectra of graphitic materials, SWNTs possess bands associated with the radial breathing mode (RBM) near 100–300 cm^−1^. The RBM mode is sensitive to *d*_t_ of an SWNT in that its frequency is inversely proportional to *d*_t_.^[^ ^23^, ^80^, ^81^ ^]^ Fantini and co‐workers^[^ ^23^ ^]^ described the energy dependence of Stokes and anti‐Stokes Raman scatterings in the 1.52–2.71 eV range of SWNTs dispersed in aqueous solution and as solid bundles. The *e*_*ii*_ and the RBM frequencies (*ω*_RBM_) were obtained for 46 different (18 *m*‐ and 28 *s*‐) SWNTs (Figure 6C,D), and (*n*, *m*) assignments were discussed based on the observed geometrical patterns in *e*_*ii*_ versus *ω*_RBM_ plots.

Rayleigh scattering spectroscopy also can be employed to analyze the (*n*, *m*) properties of SWNTs. As opposed to inelastic Raman scattering, Rayleigh scattering is elastic scattering when the size of a material is smaller than the wavelength of excitation. In particular, the scattering intensity increases as DOS increases, so the method can be used to determine the vHs of SWNTs.^[^ ^26^, ^82^, ^83^, ^84^ ^]^ As shown in Figure 6E, intense laser light irradiation of suspended SWNTs leads to scattered radiation indicated by the color corresponding to the resonance. Two separated peaks associated with the *e* ^S^_33_ and *e* ^S^_44_ transitions are observed in the Rayleigh scattering spectrum of *s*‐SWNT (top of Figure 6F). In contrast, a single broad peak corresponding to the *e* ^M^_22_ transition is present in the Rayleigh scattering spectrum of *m*‐SWNT (bottom of Figure 6F). Wang and co‐workers^[^ ^26^ ^]^ analyzed SWNTs suspended in a trench created by CVD using Rayleigh scattering spectra generated using varying excitation energies, which enabled assignment of SWNT type. Furthermore, Joh and co‐workers^[^ ^27^ ^]^ described a novel on‐chip Rayleigh imaging technique that can be employed to characterize individual SWNTs with high spatial and spectral resolution. The chiralities and electronic properties of SWNTs were verified using AFM, leading to obtain absorption and scattering cross‐section values (*σ*) which are universal indicators of light response for the excited light flux.

In addition to these approaches, scanning electron microscopy (SEM) has been utilized to distinguish between *s*‐ and *m*‐SWNTs. In one study, J. Li and co‐workers^[^ ^85^ ^]^ used voltage contrast SEM to discriminate between *s*‐ and *m*‐SWNTs. When a SWNT sample is irradiated using an electron beam in the absence of an applied voltage, *m*‐SWNT displays a much brighter SEM image than does *s*‐SWNT due to emission of higher numbers of secondary electrons from free electrons in *m*‐SWNT (left of Figure 6G). Furthermore, when plus and minus voltages are applied, the brightness of *m*‐SWNT in the SEM image dramatically changes due to its higher electrical conductivity than that of *s*‐SWNT, which does not undergo a brightness change (middle and right of Figure 6G). Thus, SEM image brightness changes can be used to distinguish SWNT electronic types. Moreover, electron diffraction patterns in TEM measurement enable precise determination of the (*n*, *m*) index of SWNTs,^[^ ^86^ ^]^ and scanning tunneling microscopy^[^ ^87^ ^]^ can be used to determine both atomic the structure and electronic DOS of SWNTs.

The responses arising from these spectroscopic methods can be expressed as *σ*. As shown in **Table** **1**, *σ* values of light absorption associated with *e* ^S^_11_ and *e* ^S^_22_ transitions^[^ ^88^, ^89^, ^90^, ^91^ ^]^ are large (≈10^−17^ and ≈10^−18^ cm^2^ C^−1^, respectively), followed by fluorescence (i.e., 10^−19^ cm^2^ C^−1^),^[^ ^88^, ^92^ ^]^ Rayleigh scattering (i.e., 10^−20^ cm^2^ C^−1^),^[^ ^93^ ^]^ and Raman scattering (i.e., 10^−22^ cm^2^ C^−1^ sr).^[^ ^94^ ^]^ Furthermore, various spectroscopic tools used for characterization of SWNTs have their individual advantages and weaknesses. For example, wavelength maxima of all SWNT types overlap, necessitating the use of deconvolution methods to separate contributions associated with each. On the other hand, although PLE measurements can be used to determine intrinsic optical transitions of each *s*‐SWNT type, it cannot be employed for *m*‐SWNT analysis. Also, while both *s*‐ and *m*‐SWNT can be accurately identified by their characteristic RBM bands, the assignments require using multiple lasers. In addition, the Rayleigh scattering method has the advantageous ability to determine SWNT types regardless of whether or not a bandgap exists, sample preparation is sophisticated and characterization requires a strong broadband light source. Finally, SEM analysis is useful for simultaneous distinction of a large array of SWNTs according to electronic type, it is limited by the fact that it can only be employed to analyze of solid samples.

**Table 1:** Advantages, weaknesses, and *σ* values of different spectroscopic methods

Methods	Advantage	Weakness	*σ* [cm^2^ C^−1^]	References
Absorption	Assignment of electronic type, chirality and bundles	Assignment of *e*_*ii*_ in ensemble sample	≈10^−17^ for *e* ^S^_11_	[88, 89, 90, 91]
			≈10^−18^ for *e* ^S^_22_	
PLE	Assignment of *e*_*ii*_ for multiple chiralities of *s*‐SWNT	Applicable only for *s*‐SWNT	≈10^−19^ for *e* ^S^_22_	[88, 92]
RRS	Assignment of electronic type and chirality	Requiring multiple laser lines	≈10^−22^ for RBM	[94]
Rayleigh scattering	Assignment of electronic type, chirality and bundles	Solid sample preparation	≈10^−20^	[93]
SEM	Assignment of electronic type	Chirality assignment	–	–

### 3.4 Photoluminescence‐Based Determination of Relative and Absolute Binding Affinities

**Figure 7:**
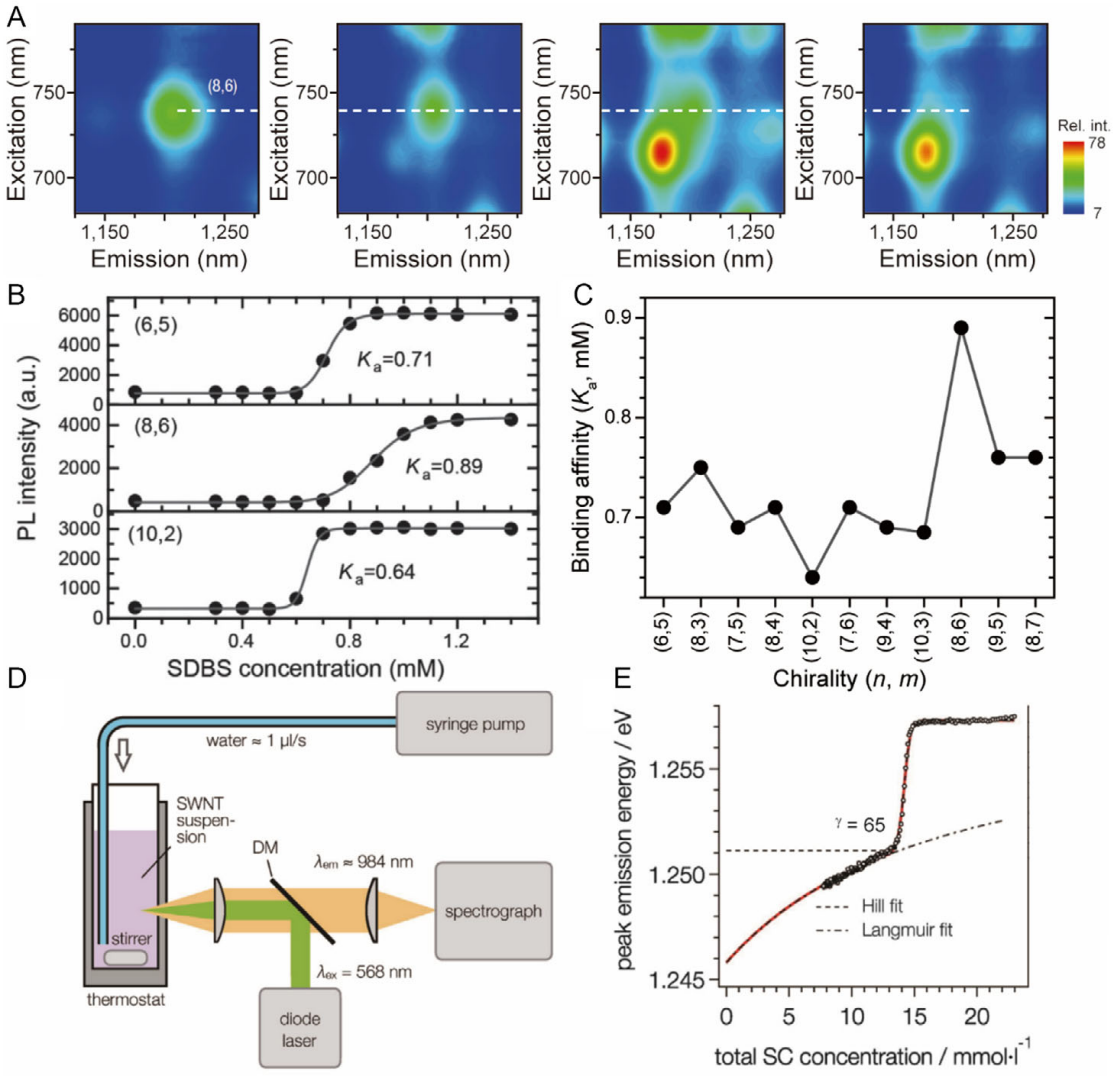
PL‐based determination of *K*_a_ for equilibrium between dispersant and SWNT. A) Changes in PL intensity and position of (8,6) SWNT upon replacement of FMN by SDBS. Reproduced with permission.^[^ ^65^ ^]^ Copyright 2008, Springer Nature. B) SDBS‐derived PL intensity traces of (6,5), (8,6), and (10,2) SWNTs versus. SDBS concentration. Gray sigmoidal curves are based on a Hill equation fitting. C) Determined *K*_a_ of 11 SWNT types. Reproduced with permission.^[^ ^96^ ^]^ Copyright 2013, Royal Society of Chemistry. D) Schematic illustration of the epifluorescence setup used for measurement of SWNT exciton PL changes during dilution experiments. E) Comparison of the step‐up in PL peak emission energy observed for SC‐dispersed (6,5) SWNTs. Reproduced with permission.^[^ ^97^ ^]^ Copyright 2016, American Chemical Society.

Among methods developed to determine binding affinity‐related equilibrium constants, *K*_a_, the most widely known is PL based codispersant titration. In this method, dispersant promoted replacement of another dispersant on the SWNT surface and is determined by monitoring changes taking place in PL intensities and wavelengths, which are then analyzed in the form of a series of PLE maps associated with codispersant concentrations. These determinations give *K*_a_ values that are relative to that of the prebound dispersant. Ju and co‐workers^[^ ^65^ ^]^ were the first to employ this method to explore dispersant replacement on SWNTs. This approach is based on the fact that PL properties of SWNTs reflect the external dielectric environment, which is altered by dispersant wrapping on the SWNT surface.^[^ ^65^, ^75^, ^95^ ^]^ As shown in the series of PLE maps in **Figure** **7**A, the initial PL intensity at long wavelengths corresponding to FMN‐(8,6) SWNT decreases as the concentration of added SDBS increases, while the PL intensity at the short‐wavelength band corresponding to SDBS‐(8,6) SWNT increases. The PL wavelength of the SWNT is blueshifted during the titration process due to the occurrence of SDBS replacement. Changes in the PL intensity brought about by increasing SDBS concentration follow aforementioned sigmoidal profiles, which differ in accordance with the type of SWNT. Oh and co‐workers^[^ ^75^ ^]^ extended this titration method to include other dispersants like SC and SDS and both absorption and PL spectroscopic techniques. Sim and co‐workers^[^ ^96^ ^]^ further revealed that the reactivity of SWNT with diazonium salt is affected by the *K*_a_ of different dispersants according to SWNT chirality (Figure 7B). In addition, the *γ* value representing dispersant cooperativity has an impact on the slope of the rise phase of the sigmoidal curve. For example, the intensity change of (8,6) SWNT, which has a lower positive *γ* value, has a more slowly rising of sigmoidal curve, whereas the curve for the PL intensity of (10,2) SWNT with a relatively higher *γ* value increases more rapidly. This is an important feature because SWNT having larger *γ* values would be useful to sort multiple SWNT types. In Figure 7C, relative *K*_a_ values as a function of SWNT type obtained by titrating FMN‐SWNT with SDBS are shown, where the *K*_a_ of FMN is largest for (8,6) SWNT. Monitoring dispersant substitution processes using PLE mapping is advantageous when peaks associated with SWNTs wrapped by two dispersants are clearly distinguishable.

While only relative SWNT binding affinities are obtained using the two‐dispersant system, the absolute binding affinity of a specific dispersant can be determined by a technique involving dilution of a SWNT dispersion, which modifies the concentrations of components in the equilibrium between the SWNT and dispersant and the dispersant–SWNT. This approach was utilized by Bergler and co‐workers^[^ ^97^ ^]^ to determine the absolute binding affinity of SC for (6,5) SWNT by evaluating changes in the PL emission energy caused by dilution‐induced desorption of SC (Figure 7D). When the SC concentration reaches the point at which SC desorbs from the SWNT surface, PL of SWNT undergoes a steep continuous change to longer wavelengths (red shift) (Figure 7F). The absolute binding affinity between SC and (6,5) SWNTs is then obtained by fitting the change in the PL position using the Hill equation (Equation (12)). The *γ* value determined for SC‐(6,5) SWNT is 65, which is higher than the aggregation number for micelle formation by the SC itself,^[^ ^98^ ^]^ indicating that the observed abrupt PL change arises from disruption of the SC supramolecular micelle structure on the SWNT surface. Also, the continuous PL redshift occurring following the rapid PL change follows the Langmuir isotherm trend, indicating that desorption of monolayer SC on the SWNT surface and bundling of the SWNT continue as dilution increases.

The PL‐based methods for determining relative and absolute binding affinities are widely used for analysis of *s*‐SWNTs that exhibit PL. In the aqueous two‐phase extraction (ATPE) topic, this method is the effective for deriving optimal extraction conditions before actual extraction, under various medium and dispersant conditions.^[^ ^99^, ^100^, ^101^, ^102^ ^]^ However, this method cannot be used to determine *K*_a_ of *m*‐SWNT which does display PL.^[^ ^61^ ^]^

### 3.5 Absorption‐Based Binding Affinity Determination

**Figure 8:**
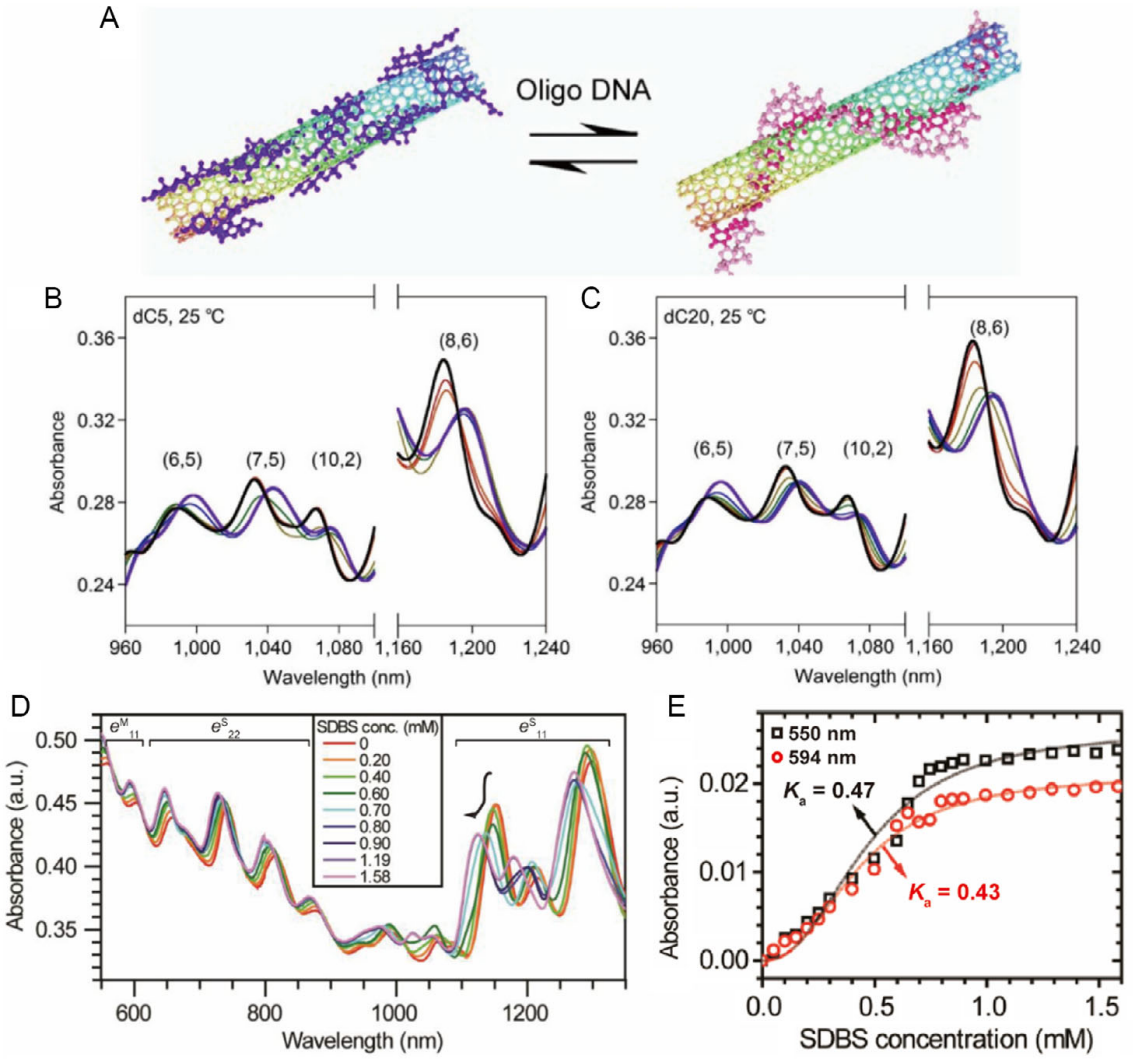
Absorption‐based determination of dispersant *K*_a_ with SWNT. A) Schematic of the SC‐SWNT and DNA‐SWNT exchange reaction. Absorption spectra of the SC‐SWNT dispersion in the absence (thick black line) and presence (colored lines) of B) dC5 and C) dC20. Reproduced with permission.^[^ ^103^ ^]^ Copyright 2012, Springer Nature. D) Absorption spectra of FMN‐wrapped SWNTs titrated with SDBS. E) Sigmoidal absorption transitions of the absorption band of the *m*‐SWNT positioned at 550 nm (black squares) and 593 nm (red circles) from the SDBS‐wrapped SWNT positions. Black and red curves are fitted using the Hill equation. Reproduced with permission.^[^ ^75^ ^]^ Copyright 2012, Springer Nature and 2013 American Chemical Society.

Unlike the PL‐based *K*_a_ determination methods described earlier, absorption‐based protocols are applicable even for *m*‐SWNTs that do not display PL. Kato and co‐workers^[^ ^103^ ^]^ used an absorbance‐based titration process to calculate *K*_a_ for equilibrium involving ss cytosine oligomers (dCn). As shown in **Figure** **8**A, ss cytosine oligomers (dCn, where n is the number of cytosine monomers) replaces SC on the SWNT surface. Binding of the dCn causes a simultaneous change in the absorption intensity and wavelength (Figure 8B,C), that is dependent on the length of the DNA chain and temperature. In the case of cytosine oligomers smaller than 8mer, replacement is driven by enthalpy.

The advantage of the absorption spectroscopy‐based binding affinity determination method is that *K*_a_ can be determined for *m*‐SWNTs. One example of this feature is found in a study by Oh and co‐workers,^[^ ^75^ ^]^ in which *K*_a_ was determined for *e* ^S^_11_ and *e* ^S^_22_ of *s*‐SWNT as well as *e* ^M^_11_ of *m*‐SWNT by analyzing absorption spectra (Figure 8D) obtained by titrating FMN‐SWNT with SDBS. Changes in the 550 and 594 nm absorption bands corresponding to the *e* ^M^_11_ ensemble of *m*‐SWNT were inserted into Equation (12) to calculate *K*_a_. Figure 8E shows the sigmoidal fit of the data, showing that *K*_a_ for the equilibrium between *s*‐SWNT and FMN is two times higher than that for *m*‐SWNT and FMN. It should be noted however that, when various types of SWNTs are present, absorption spectra congestion could occur, making it impossible to identify distinctive types,^[^ ^104^ ^]^ and optical deconvolution to assign SWNT chiralities is not sufficiently accurate due to overlapped optical transition and electrons–phonons.

### 3.6 Other Methods to Determine Binding Affinity

**Figure 9:**
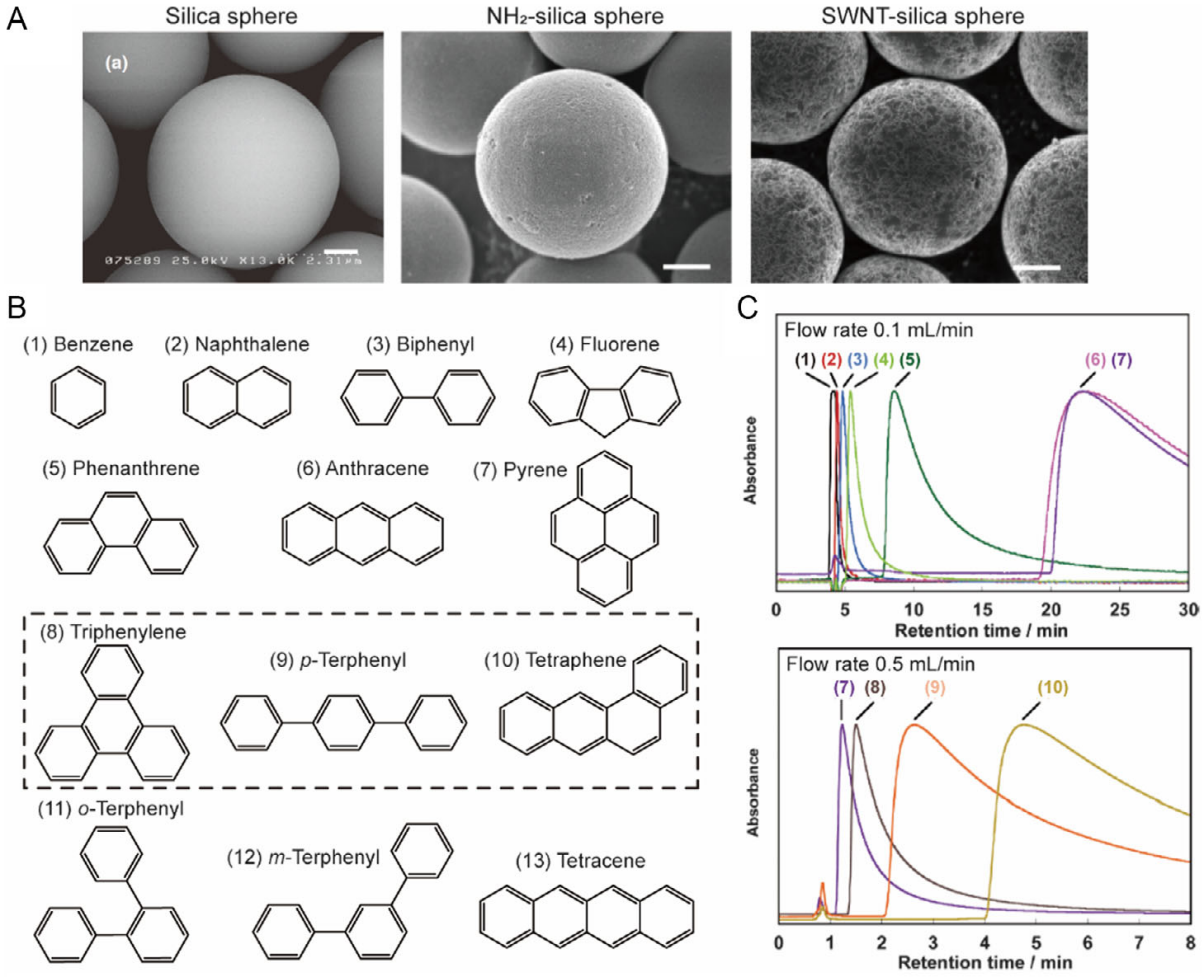
HPLC‐based binding affinities between SWNT and aromatic molecules. A) SEM images of bare silica sphere, NH_2_‐silica sphere, and SWNT‐coated silica sphere. Scale bar: 1 μm. Reproduced with permission.^[^ ^232^ ^]^ Copyright 2011. The Chemical Society of Japan. B) Chemical structures of PAHs. Structures in dashed box indicate molecules with the same number of carbons (i.e., 18 atoms). C) Chromatograms of various PAHs using a SWNT‐coated silica column and tetrahydrofuran as eluent. Reproduced with permission.^[^ ^105^ ^]^ Copyright 2011, Royal Society of Chemistry.

The PL‐ and absorption‐based methods enable determination of relative and absolute binding affinities using dispersant titration and dilution. However, other approaches utilizing chromatography and nuclear magnetic resonance (NMR) spectroscopy can be employed to obtain information about interactions between SWNT surfaces and small molecules. High‐performance liquid chromatography (HPLC) was utilized to determine the *K*_a_ values associated with binding of polycyclic aromatic hydrocarbons (PAHs). Yoo and coworkers^[^ ^105^ ^]^ assessed interactions between SWNTs and PAHs using SWNT‐coated silica spheres as the stationary phase in an HPLC system. For this purpose, SWNT‐coated silica was prepared by applying SWNTs to NH_2_‐terminated silica spheres (**Figure** **9**A). The *K*_a_ values for equilibria between SWNTs and PAHs were then obtained by analyzing retention time data (Figure 9B). Peaks in chromatograms generated using SWNT‐coated silica columns have retention times that become longer as the number of aromatic rings (N_Ar_) in the PAH increases (Figure 9C). For molecules with the same N_Ar_, those comprise polyphenyls (i.e., biphenyl and *p*‐terphenyl) have longer residence times than do those composed of polyacene moieties (i.e., naphthalene and anthracene) because the effective area for overlap with the SWNT is larger in the linearly bonded polyphenyl systems. In addition, linear fused acenes like tetracene have larger effective overlap areas than their nonlinear counterparts like triphenylene), a trend that is also followed for polyphenyl substances such as *o*‐, *m*‐, and *p*‐terphenyls.

**Figure 10:**
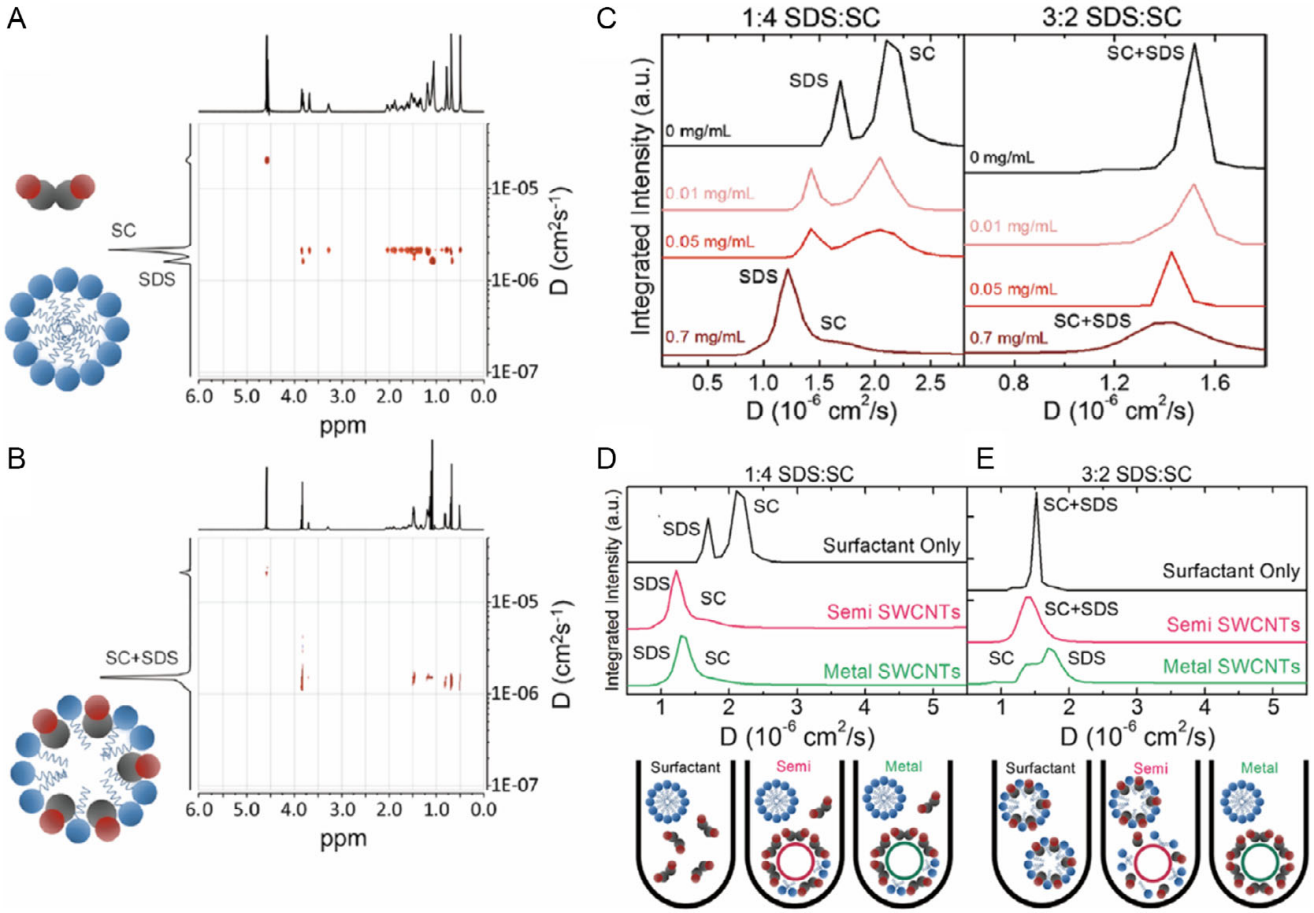
NMR spectroscopy‐based determination of dispersant organization on the SWNT surface. 2D DOSY NMR spectra of A) 1:4 and B) 3:2 SDS:SC samples. C) Integrated ^1^H NMR intensity versus *D* for dispersant only, *s*‐ and *m*‐SWNT in D) 1:4 and E) 3:2 SDS:SC ratio. (Bottom of (E)) The corresponding schematic of dispersant micelle with or without SWNT. Reproduced with permission.^[^ ^106^ ^]^ Copyright 2013, American Chemical Society.

The NMR spectroscopy technique known as 2D diffusion ordered spectroscopy (DOSY)^[^ ^106^ ^]^ has been employed to analyze interactions between dispersant molecules and nanostructures including SWNTs.^[^ ^107^, ^108^, ^109^ ^]^ The interactions are reflected in the disappearance, shifting, and broadening of ^1^H and ^13^C NMR peaks. Shastry and co‐workers^[^ ^106^ ^]^ determined diffusion coefficient (*D*) differences between SC and SDS on the SWNT surface using 2D DOSY NMR. The 2D DOSY NMR spectrum of a 1:4 (**Figure** **10**A) mixture of SDS and SC in the absence of the SWNT contains peaks for each dispersant at different *D* values, which show that each dispersant forms its own micelle. However, when the SC concentration ratio is 3:2 (Figure 10B),^[^ ^110^ ^]^ a single peak is present in the 2D DOSY NMR spectrum which is associated with a micelle containing both SDS and SC. When *m*‐SWNT is present in 1:4 SC:SDS solution, the two peaks combine into one peak at a lower *D* value (Figure 1C), indicating that a mixed micelle structure forms on the SWNT surface. In contrast, when *m*‐SWNT is added to 3:2 SC:SDS solution, the one peak in the spectrum associated with the mixed micelle is split into two peaks while when *s*‐SWNT is added the single peak is retained in the spectrum (Figure 10D). These observations led to the proposal that addition of *m*‐SWNT to the 3:2 SC:SDS mixture causes SC molecules in the mixed micelle to selectively adsorb on the *m*‐SWNT surface and the remaining molecules of SDS form pure non‐SWNT containing micelles.

## 4 Methods of SWNT Separation via Control of the Dispersant Binding Affinity

### 4.1 DNA‐Based Ion Exchange Column Chromatography

The nucleic acids DNA and RNA are biopolymers that carry genetic information of organisms. Double‐stranded DNA (dsDNA) consists of two nucleic acid chains arranged in a double‐helix structure. Each chain consists of molecular building blocks called nucleotides which comprise our bases including adenine (A), thymine (T), guanine (G), and cytosine (C). In the helical structure, the sugar–phosphate groups are oriented on the outside of the helix and the bases located on the inside are hydrogen bonded with matching bases on the other chain. Sequence modification of DNA is widely utilized in the material sciences such as DNA origami^[^ ^111^ ^]^ and linker^[^ ^112^ ^]^ to control binding energies between the chains that are governed by the number of hydrogen bonds between the bases. In 2003, Zheng and co‐workers^[^ ^113^ ^]^ described the phenomenon of DNA‐assisted SWNT dispersion and sorting, in which SWNTs are effectively dispersed in water by sonication in the presence of ssDNA. This group also showed that the DNA‐based SWNT dispersion can be used to separate electronic type different SWNTs using ion exchange chromatography (IEC), which takes advantage of differences in electrostatic interactions with stationary phases. *m*‐SWNT, which has a higher polarizability than *s*‐SWNT, is less effective in dissipating negative charges on ssDNA. Due to its lower negative charge, ssDNA‐complexed *m*‐SWNT interacts to a lesser extent with the cationic resin and, thus, it has smaller retention time on the column than ssDNA‐complexed *s*‐SWNT. In addition, Zheng and co‐workers^[^ ^67^ ^]^ found that sorting of ssDNA‐wrapped SWNT depends on the DNA sequence. A systematic study of a ssDNA library revealed that the electrostatic interactions in the DNA–SWNT hybrid depend on *d*_t_ and the electronic properties of SWNT. As a result of these polarity differences, it is possible to use this technique to separate *s*‐SWNT based on their *d*_t_ values.

**Figure 11:**
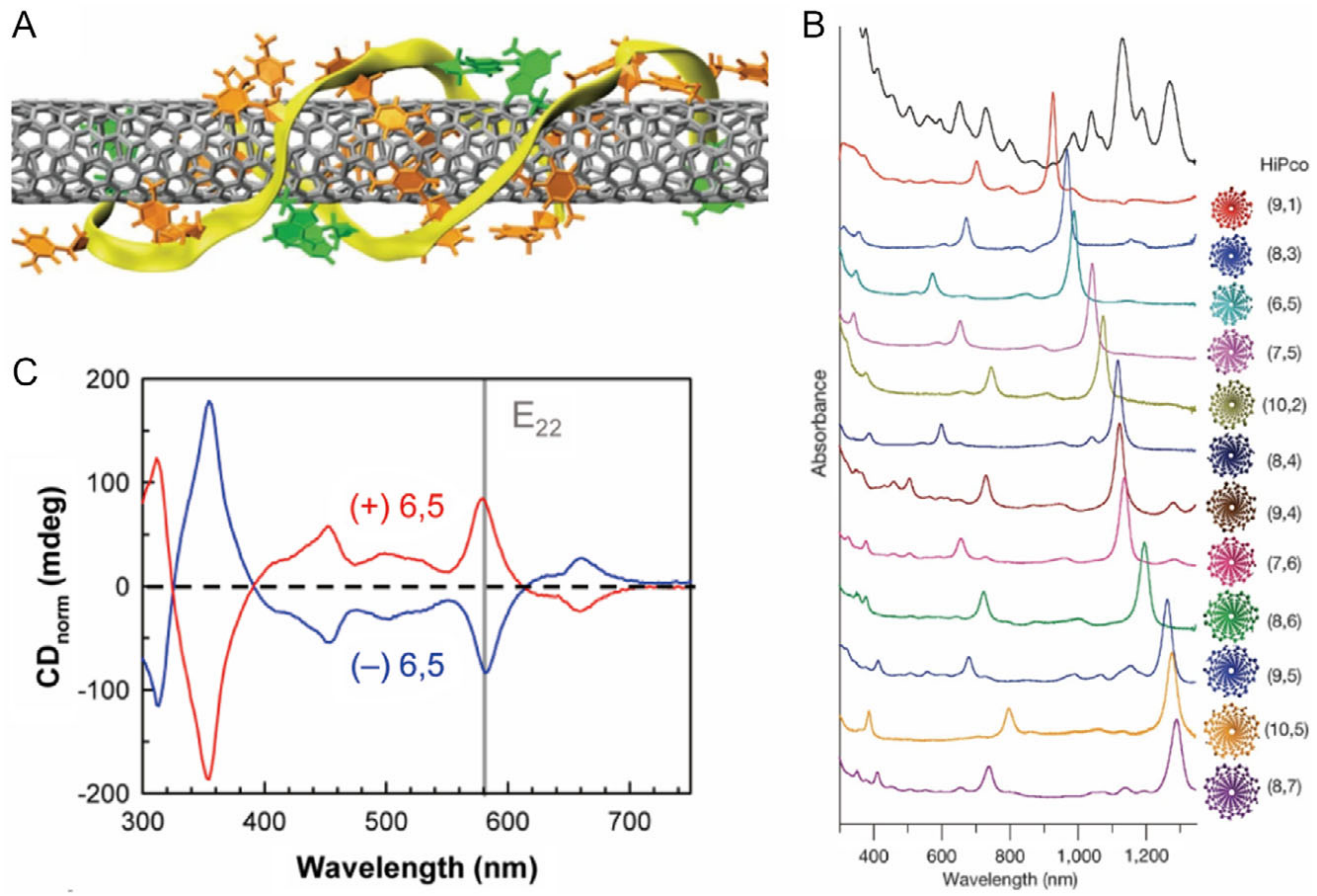
DNA‐based sorting of SWNT according to type and handedness. A) A ssDNA barrel on a (8,4) SWNT formed by rolling up a 2D DNA sheet composed of two hydrogen‐bonded antiparallel ssDNA. Color code: orange, T; green, A; yellow ribbons, backbones. B) UV–vis–NIR absorption spectra of 12 purified *s*‐SWNTs (ranked according to the measured *e* ^s^_11_ absorption wavelength) and the starting SWNT mixture. The structure of each purified SWNT species and their (*n*, *m*) notation are given on the right side of the corresponding spectrum. Reproduced with permission.^[^ ^114^ ^]^ Copyright 2009, Springer Nature. C) CD spectra of ssDNA‐(6,5) enantiomer hybrids selectively isolated using a multistage extraction process. The *P*‐(6,5) and *M‐*(6,5) enantiomers have nearly equal intensities and opposite signs. Reproduced with permission.^[^ ^115^ ^]^ Copyright 2016, American Chemical Society.

In 2009, Tu and co‐workers^[^ ^114^ ^]^ described a method that enables the separation of single types of *s*‐SWNT utilizing 20 different ssDNA sequences. The ssDNA recognition sequences contain periodic purine–pyrimidine patterns that form hydrogen bonds in a 2D manner that leads to selective folding into well‐ordered 3D barrels on SWNT surfaces (**Figure** **11**A). An SWNT wrapped with an aligned ssDNA barrel is eluted from the IEC column faster. In this manner, 12 SWNT types were sorted by controlling the ssDNA sequence (Figure 11B). In addition, sorting based on handedness was achieved by applying an ATPE method which is described (Figure 11C).^[^ ^115^ ^]^

DNA has a selective wrapping structure on the surface of various SWNTs with different *d*_t_ and chiral angles depending on the type and length of the base sequence. Excellent SWNT chirality recognition based on the DNA base sequence, combined with IEC and ATPE separation methods, showed the separation of multiple species of single chirality.^[^ ^116^ ^]^ Although various combinations enable sorting of specific types, preparation of the desired ssDNA sequences is relatively more expensive than that of dispersants. In order to save the cost and time of finding ssDNA suitable for separation, a study was presented to predict ssDNA suitable for separation using machine learning.^[^ ^117^ ^]^ From an application perspective, the strongly bound ssDNA on the SWNT surface is difficult to remove.

### 4.2 Density Gradient Ultracentrifugation

Density gradient ultracentrifugation (DGU) is commonly used to isolate biological substances such as proteins, RNA, and DNA.^[^ ^118^ ^]^ DGU sorts biological molecules according to differences in their buoyant density (*ρ*). In this technique, dispersed mixtures are subjected to ultracentrifugation typically at >100 000 gravitational acceleration (*g*) in a density gradient medium. As a result, substances are sorted into layers where their *ρ* values match that of the medium. Iodixanol is typically used as density gradient medium for sorting dispersant‐wrapped single SWNT species but other media (i.e., cesium chloride^[^ ^119^ ^]^ and sucrose^[^ ^120^ ^]^) have been utilized. The density of a dispersant‐wrapped SWNT depends on dispersant type (i.e., *ρ* of SC‐SWNT:^[^ ^121^ ^]^ 1.05–1.11 g cm^−3^, *ρ* of DNA‐SWNT:^[^ ^122^ ^]^ 1.11–1.17 g cm^−3^, and *ρ* of FMN‐SWNT:^[^ ^123^ ^]^ 1.15–1.25 g cm^−3^). The *ρ* value of a commercially available iodixanol solution (i.e., 60 w./v%) is 1.320 g cm^−3^, and it can be accurately controlled to sequential values through dilution with water. However, the high costs of iodixanol and issues with residues in sorted *s*‐SWNTs are clear limitations.

**Figure 12:**
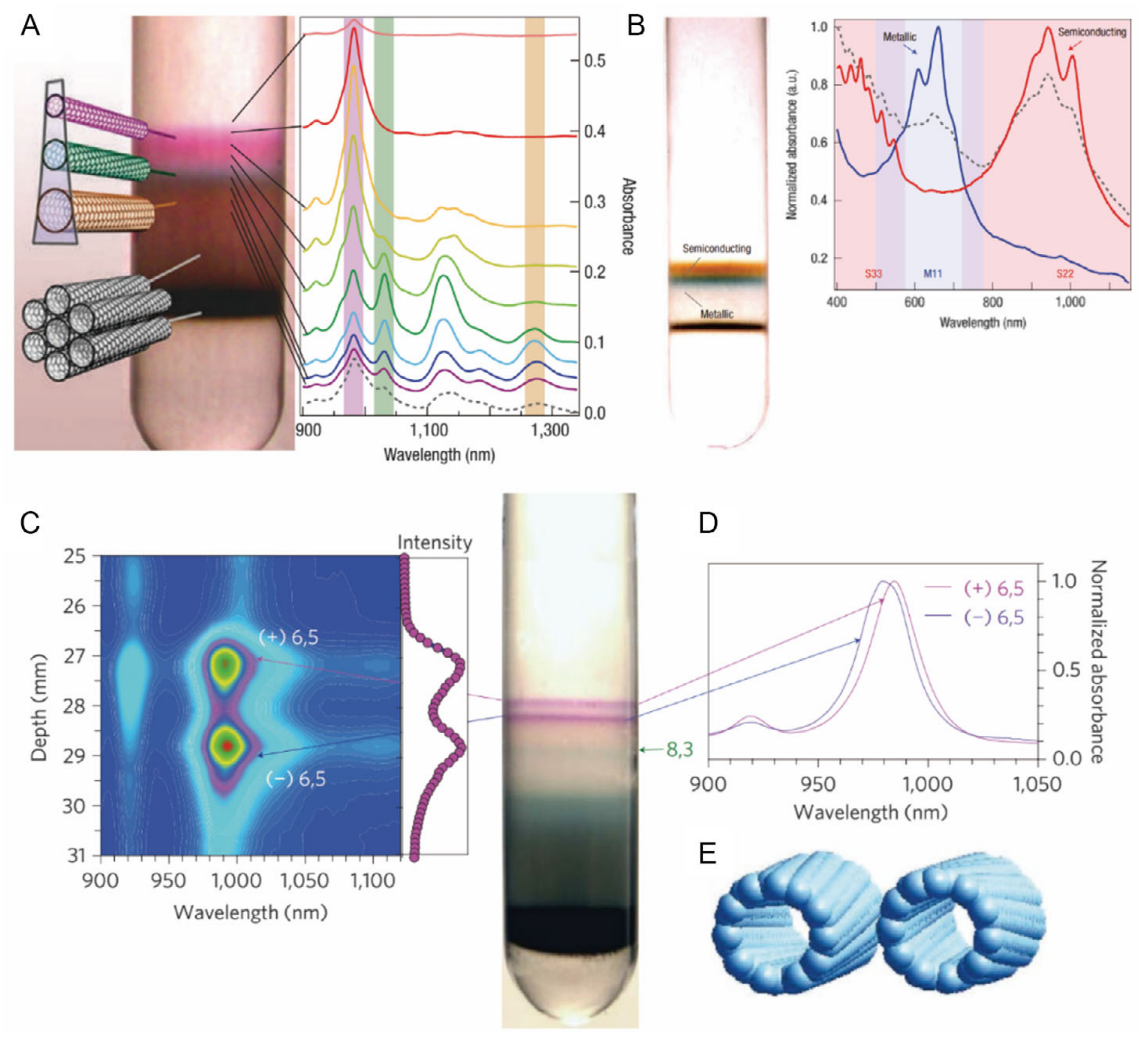
DGU‐based sorting of SWNT according to *d*_t_, electronic type, and handedness. A) Schematic (left) and layer‐specific absorption spectra (right) from the DGU method using SC. Mixed SDS/SC‐based SWNT sorting. B) *s*‐ and *m*‐SWNT enrichment using DGU. Reproduced with permission.^[^ ^124^ ^]^ Copyright 2006, Springer Nature. C) (Right) (6,5) enantiomer separation in density gradient medium and (left) depth‐profiled PL map. D) Absorption spectra according to DGU depth showing optical transition differences of spatially resolved (6,5) enantiomers. E) Schematics of (6,5) enantiomers (left: *M*‐(6,5), right: *P*‐(6,5)). Reproduced with permission.^[^ ^121^ ^]^ Copyright 2010, Springer Nature.

In 2005, Arnold and co‐workers^[^ ^122^ ^]^ found that sorting SWNTs wrapped by ssDNA can be performed by utilization of the DGU method. However, the DNA‐wrapped SWNT has low stability during repetitive centrifugation needed to improve the purity of SWNT type. Also, the difficulties associated with the complete removal of DNA after sorting and the cost of DNA are limitations. Moreover, this approach is not useful for sorting according to SWNT electronic type. In 2006, Arnold and co‐workers^[^ ^124^ ^]^ introduced modification of the DGU procedure that utilizes SC and SDS to effectively sort SWNTs according to *d*_t_ and electronic type. The results of additional studies suggest that separation in this system stems from *ρ* differences that are determined by the dispersant and associated hydration layer on the SWNT surface.^[^ ^125^, ^126^ ^]^ For example, SWNTs with smaller *d*_t_ values have low *ρ*, which locates in upper layers of density gradient media. The type of dispersant affects the separation process. For example, SWNTs dispersed by SDBS or SDS, common anionic alkyl amphiphiles, cannot be not clearly separated.^[^ ^124^ ^]^ On the other hand, SWNTs dispersed using SC are effectively sorted with a tendency for those having greater *d*_t_ values to have greater *ρ* values (**Figure** **12**A). This phenomenon originates from the fact that SC and other bile salt derivatives (i.e., DOC and TDOC) have rigid and planar steroid structures which lead to stronger adsorption on the SWNT surface.

Further modification of *ρ* of bile salt‐wrapped SWNTs is possible by introducing another dispersant. The loosely packed structure of SDS on SWNTs can be used to fine tune hydrophobicity. *m*‐ and *s*‐SWNTs were sorted utilizing SDS/SC mixed‐dispersant system (Figure 12B),^[^ ^124^ ^]^ owing to CH–*π* interactions induced by charge transfer between the dispersant and *m*‐SWNT.

In 2009, Green and co‐workers^[^ ^127^ ^]^ were able to enrich the handedness of SWNTs employing a single SC dispersant, and in 2010 Ghosh and co‐workers^[^ ^121^ ^]^ succeeded in effectively sorting several SWNTs according to both handedness and type using mixed dispersants (i.e., SDS and SC) (see Figure 12C–E for (6,5) SWNT handedness separation). Due to the multiple stereogenic centers present in SC, it wraps *P‐* and *M*‐SWNT in a slightly different manner Furthermore, when SDS is mixed with SC, the dispersant supramolecular structure is slightly changed due to competitive absorption on the SWNT surface by SDS and SC. This minute difference causes the SWNT enantiomers to locate in distinct layers with different *ρ*. Although the introduction of other polymeric dispersants to enhance separation efficiency has been made, no combination has been reported to surpass the sorting efficiency of bile salt/SDS.^[^ ^128^ ^]^

The combined observations demonstrate that DGU is a powerful method for sorting single‐species SWNTs. Moreover, it is an effective way to remove SWNT bundles, which are utilized as the initial step in other sorting methods. Finally, The DGU method has been applied in manufacturing SWNT dispersions despite the need for high‐cost equipment and scaling up difficulties.^[^ ^129^, ^130^ ^]^ However, DGU has limitations that make it difficult to scale up due to long centrifugal time and high‐cost equipment. Additionally, in the process of obtaining the sorted SWNT layer, it is difficult to completely retrieve only the desired separated layer and to remove the medium remaining in the dispersion after separation. The medium removal process can induce rebundle of SWNTs.

### 4.3 Poly‐Fluorenes

**Figure 13:**
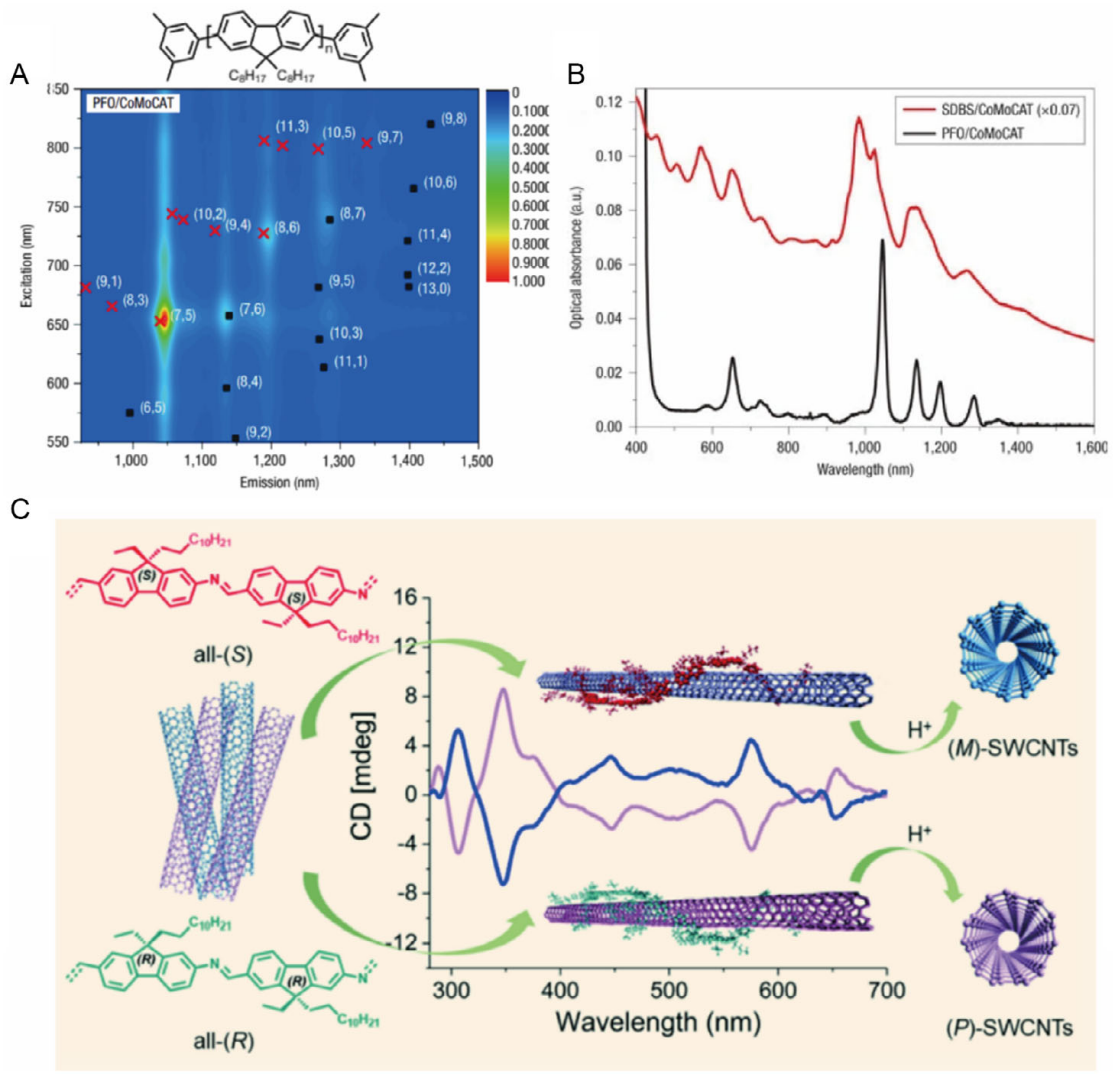
PFO‐based sorting of near‐armchair‐type SWNT. A) PLE map of prominent (7,5) peak and other near‐armchair SWNTs. B) UV–vis–NIR absorption spectra of different dispersants interacting with SWNTs that have average *d*_t_ values lower than 1 nm. Reproduced with permission.^[^ ^132^ ^]^ Copyright 2007, Springer Nature. C) Molecular structures of all‐(R) and all‐(S) chiral PFOs for handedness sorting of *s*‐SWNT. Reproduced with permission.^[^ ^135^ ^]^ Copyright 2021, American Chemical Society.

Polymeric dispersants, which stabilize SWNTs due to entropy changes of the polymers themselves,^[^ ^131^ ^]^ can have interesting effects on SWNTs. Especially interesting are *π*‐conjugated polymer–SWNT constructs that have unique optoelectronic features. In 2007, Nish and co‐workers^[^ ^132^ ^]^ (**Figure** **13**A) and later Chen and co‐workers^[^ ^133^ ^]^ reported that poly(fluorene) (PFO), which is a rigid rod *π*‐conjugated polymer, selectively disperses near armchair‐type *s*‐SWNT. Nish and co‐workers^[^ ^132^ ^]^ reported that rigid rod aromatic polymers dramatically enhance the formation of high‐purity *s*‐SWNT dispersions as a consequence of selectivity created by their supramolecular structures. Moreover, as compared to SWNTs dispersed in micellar dispersants, dispersions of PFO with SWNTs having small *d*_t_ values display nearly no background absorption and narrow bandwidths in their absorption spectra (Figure 13B).

In addition, chiral center in the main chain of PFO monomer was utilized to separate SWNT handedness. Akazaki and co‐workers^[^ ^134^ ^]^ were the first to utilize a binaphthol as a copolymer in PFO to create a dispersant that separates *M‐* and *P‐*SWNTs through one‐pot sonication. Furthermore, Xu and co‐workers^[^ ^135^ ^]^ reported that enantiomerical pure chiral PFO, containing stereogenic centers linked to chain length with different lengths (i.e., ethyl and dodecyl groups), can be utilized to sort SWNTs according to handedness (Figure 13C). These chiral polymers selectively recognize *M‐* or *P*‐SWNT in small *d*_t_ SWNTs. Other investigations are probing the selectivities of chiral, alkyl chain, and atomic ring‐containing polymeric PFOs for separating SWNT with opposite handedness.^[^ ^136^, ^137^, ^138^ ^]^ In addition to altering the structure of PFO, separation efficiency can be enhanced using a mixed solvents^[^ ^139^ ^]^ or mixing PFO dispersants tailored to each chirality.^[^ ^140^ ^]^ Moreover, a high‐purity monochiral separation is achieved by redispersion with other PFO dispersants after the initial dispersant removal, leveraging the differences in chirality selectivity among PFO dispersants.^[^ ^141^ ^]^

Advantages of PFO‐based *s*‐SWNT sorting include its simplicity and ability to produce high‐purity SWNTs not containing carbonaceous impurities. In addition, the conducting nature of PFOs facilitates the use of their SWNT complexes in optoelectronic devices such as organic light‐emitting diodes.^[^ ^142^ ^]^ Due to the excellent dispersion properties of PFOs, they are being applied to many PFO‐based SWNT sorting applications, but it should still be pointed out that they have several disadvantages: PFO has high selectivity for *s*‐SWNTs, making it easy to separate according to electronic type.^[^ ^143^ ^]^ Additionally, *d*_t_ and chirality selectivity can be effectively controlled by changing the aromatic ring and alkyl chain that make up the PFO polymer.^[^ ^144^ ^]^ However, due to the disorder of the polymer itself, the single chirality that can be separated using PFO derivatives is very limited, and obtaining the backbone structure conditions of PFO with single chirality, selectivity remains a difficult task.^[^ ^143^, ^145^ ^]^ In terms of application, PFO has the problem of high cost compared to other SDS and bile salt dispersants. In addition, for some applications, removal of the dispersant is required after extraction, but PFO has the disadvantage of being difficult to remove. To overcome these shortcomings, we are researching ways to devise PFO^[^ ^146^ ^]^ that can easily depolymerize PFO in postprocessing or to degrade the polymer so that it dissolves well in solvent.^[^ ^147^ ^]^

### 4.4 Porphyrins

**Figure 14:**
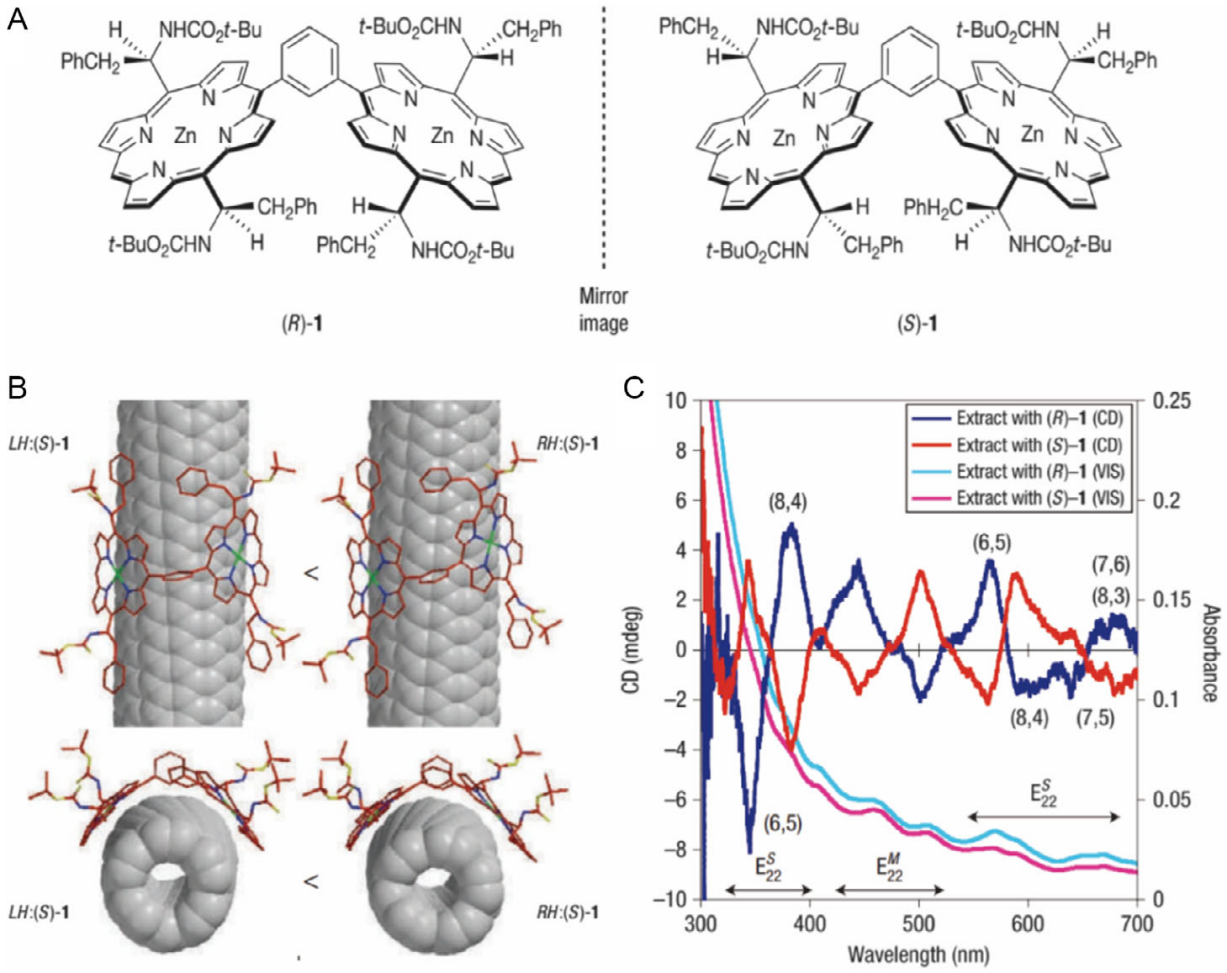
Diporphyrin‐based sorting of SWNT handedness. A) Chiral nanotweezers that are used to separate *P*‐ and *M*‐enantiomers of chiral SWNTs. B) Schematic of the structure difference and selectivity of *P*‐ and *M*‐SWNTs with a chiral diporphyrin. C) CD and absorption spectra of SWNT enantiomers separated using chiral porphyrin tweezer followed by SDBS‐promoted unwrapping. Reproduced with permission.^[^ ^50^ ^]^ Copyright 2007, Springer Nature.

Because porphyrins have high affinities with C60,^[^ ^148^, ^149^ ^]^ it is expected that they would form complexes with SWNTs. In fact, porphyrins have been employed to separate *s*‐SWNTs.^[^ ^150^ ^]^ In 2007, Peng and co‐workers^[^ ^50^ ^]^ showed that the enantiomers of a chiral SWNT could be separated using the two enantiomers of a chiral zinc diporphyrin derivative. The synthesized chiral porphyrins (*R*)‐1 and (*S*)‐1 (**Figure** **14**A) consist of two benzene ring‐bridged porphyrin rings in the form of a nanotweezer containing four stereogenic centers. The nanoconstructs created between these enantiomeric porphyrins and SWNT are stabilized mainly by *π*–*π* and CH–*π* interactions. The results of computational studies indicate that complexed state of (*S*)‐1 and *P*‐(6,5) is more stable than that of (*S*)‐1 with *M*‐(6,5) by 0.32 kcal mol^−1^ (see the structural differences between complexes formed between the diporphyrin enantiomers and each SWNT enantiomer in Figure 14B). This binding energy difference is smaller than thermal energy, *k*_B_ *T* where *k*_B_ is the Boltzmann constant and *T* the absolute temperature. However, the cumulative stabilization brought about by binding multiple chiral diporphyrins to form a supramolecular complex is expected to create a much larger binding energy difference that enhances sorting of the SWNT enantiomers. The CD profiles of the (*R*) and (*S*) enantiomers of the porphyrin complexed with the SWNT enantiomers display symmetric bisignate Cotton effects. In addition, the complete SWNT enantiomers produced by removal of diporphyrin from the complexes through replacement by SDBS still exhibit those complementary CD signals (Figure 14C), confirming separation according to handedness.

Selectivity for separation according to *d*_t_ and *θ* was achieved by modifying the bridging component that connects porphyrin rings in the diporphyrins.^[^ ^53^, ^151^, ^152^ ^]^ The *d*_t_ and *θ* ranges of SWNTs that can be separated depend on the nature (i.e., type, length, and functional group) of spacer and bridging groups which affect the dihedral angle between the porphyrin rings.

Although the diporphyrin nanotweezer approach is appropriate for sorting of handedness, its inability to produce single enantiomers with high purities is a limitation. Diporphyrin nanotweezer has excellent handedness separation capability due to the chiral center in the molecular structure. However, due to the limitations of the tweezer structure such as larger footprint per SWNT, there is no effective difference in binding affinity according to chirality, making it difficult to separate a single chirality. Additionally, the difficulty in synthesizing diporphyrin poses a problem in studying the separation of SWNTs according to various structures.

### 4.5 Flavins

**Figure 15:**
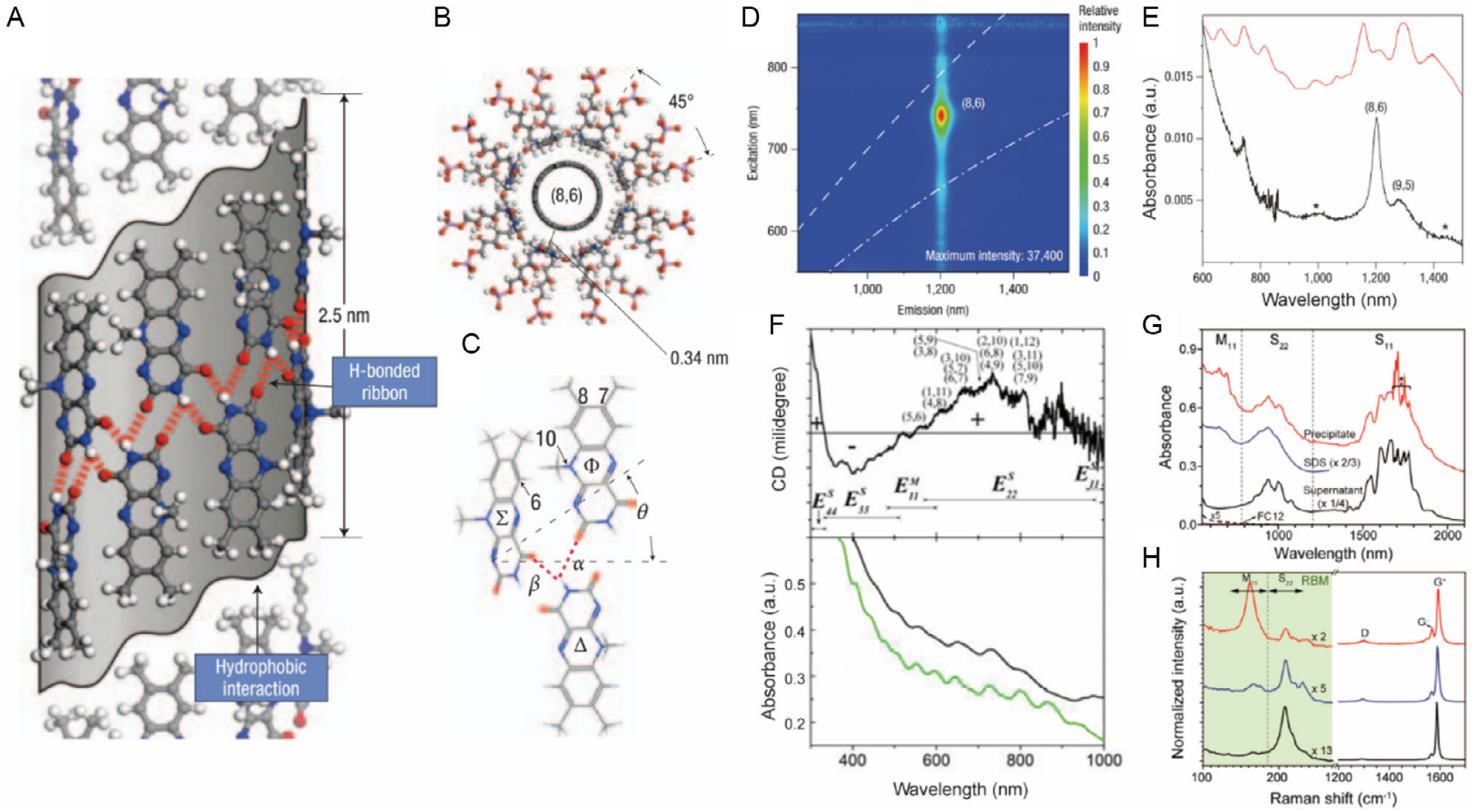
Flavin‐based sorting of SWNT according to electronic type, chirality. and handedness. A) Top view of isoalloxazine moieties wrapped in an *8*_1_ helical pattern. B) The *d*‐ribityl phosphate side groups of FMN provide aqueous solubilization. C) Hydrogen bonding (*α* and *β*) and steric (6 and 10 positions) considerations of the helical repeat pair (i.e., (Φ/Δ) or (Δ/Σ)). Enrichment of the (8,6) nanotube using selective SDBS replacement of FMN, and addition of sodium chloride to salt in the SDBS‐replaced nanotubes. D) PLE map of the (8,6) nanotube in the salted‐out supernatant. Broken white lines indicate phonon modes of SWNT. E) UV–vis–NIR spectra of the corresponding salted‐out supernatant (black solid line), compared with the initial FMN‐dispersed HiPco sample (red solid line). Asterisks indicate phonon mode absorption of (8,6) SWNTs. Handedness sorting of SWNT. Reproduced with permission.^[^ ^65^ ^]^ Copyright 2008, Springer Nature. F) CD and UV–vis–NIR spectra following FMN replacement with SDBS (black curve). SDBS‐dispersed HiPco SWNTs were used as a control (green curve). Enrichment of *s*‐ and *m‐*PSWNTs dispersed by FC12. Reproduced with permission.^[^ ^155^ ^]^ Copyright 2012, American Chemical Society. G) Absorption spectra of supernatant (black) and precipitate (red) of FC12‐PSWNT, along with that of supernatant (blue) of SDS‐PSWNT. The residual FC12 absorption tail (dotted line) having the same concentration as the dispersion is magnified on top of the initial spectrum (magenta). H) Raman RBM and G band spectra of PSWNT on 285 nm‐thick SiO_2_/Si substrate, excited using a 785 nm laser. Reproduced with permission.^[^ ^153^ ^]^ Copyright 2016, American Chemical Society.

Methods using polymer dispersants such as ssDNA and PFO although enabling selective sorting of SWNTs are limited by the difficultly in completely removing the dispersant adsorbed on the surface of SWNTs to produce materials that have excellent physical properties. In an effort aimed at avoiding this problem, Ju and co‐workers^[^ ^65^ ^]^ dispersed SWNTs using a flavin derivative which can be easily removed and replaced with other dispersants. Among the flavin derivatives, the most studied is FMN, which is composed of an isoalloxazine ring and contains a ribityl phosphate side chain (**Figure** **15**A). FMN is stably adsorbed to the SWNT surface *π*–*π* interactions between the *π*‐conjugated isoalloxazine ring and graphene wall of the SWNT. In addition, adjacent flavins on the SWNT surface participate in quadruple hydrogen bonding to create a unique helical supramolecular structure. The *d*‐ribityl phosphate side chains of FMN convey an anionic repulsion property to the SWNT dispersion. (Figure 15B,C)

In 2008, Ju and co‐workers^[^ ^65^ ^]^ found that dispersal of SWNTs using FMN followed by dispersant substitution can be used to produce (8,6)‐enriched SWNTs. As described earlier, FMN on SWNT side walls is replaced using higher‐than‐certain concentrations of SDBS. In this effort, it was found that SDBS promoted displacement of FMN‐wrapped SWNTs which led to enrichment of (8,6) SWNTs wrapped by FMN, as its larger *K*_a_ remains intact. Thus, when NaCl was added to the dispersion, all types of SWNTs were dispersed in SDBS precipitate, leaving only (8,6) SWNT in the supernatant (Figure 15D,E).

In 2009, Ju and co‐workers^[^ ^66^ ^]^ reported that the synthesized *N*‐dodecyl isoalloxazine (FC12) can be employed to disperse SWNTs in aromatic solvents. Like those formed using PFOs, dispersions generated using FC12 display narrow spectral bandwidths and minimal background originating from SWNT bundles and carbonaceous impurities. In 2016, Park and co‐workers^[^ ^153^ ^]^ along with other groups^[^ ^154^ ^]^ developed a simple one‐step FC12‐based protocol to isolate *s*‐ and *m‐*SWNT with high purity. In this process, it was found that treatment of the mixture of FC12‐complexed *s*‐ and *m‐*SWNT with SDS enabled separation of *m‐*SWNT containing dispersion and *s*‐SWNT containing supernatant that displays no absorption and RBM Raman peaks that correspond to *m‐*SWNT when compared to the control SDS‐SWNT (Figure 15F,G). The obtained high‐purity *s*‐SWNT was utilized to form a thin‐film transistor that has excellent electrical properties.

Overall, handedness enrichment of an SWNT ensemble was also achieved using FMN. Ju and co‐workers^[^ ^155^ ^]^ reported that sorting of the *M‐*SWNT ensemble takes place through formation of a hydrogen bonded FMN helix structure. Theoretical calculation showed that the 2′‐OH group of the *d*‐ribityl phosphate side chain on *N*‐10 of FMN electrostatically interacts preferentially with the adjacent uracil moiety, creating a relatively stable *P*‐helical FMN structure. As a result of preferential interaction of *P*‐helical FMN with *M*‐SWNTs, the FMN helix selectively *M‐*SWNTs first using the dispersion process. The enrichment of *M‐*SWNTs is indicated by the observed (+, −, +) sign order for e^S^_44_, e^S^_33_, and e^S^_22_, respectively, in the CD (Figure 15H).

The properties of SWNTs sorted using flavin derivatives^[^ ^156^, ^157^, ^158^ ^]^ are being explored for many purposes. Many chemical modifications of flavin derivatives in various positions^[^ ^157^ ^]^ allow to disperse SWNT with stability. For example, it was found that highly individualized SWNT dispersions display stoichiometric enrichments that are governed by the concentration of FC12. Choi and co‐workers^[^ ^95^ ^]^ observed that FC12 and FMN dispersal is dependent on the dielectric constants of the medium, and FC12‐SWNT displays solvatochromism. In addition, in studies of flavin‐containing self‐assembled monolayers (SAMs) on substrates, Park and co‐workers^[^ ^159^ ^]^ manufactured an SWNT self‐assembly system that sows *d*_t_ dependence and *m*‐SWNT selectivity. The developed flavin‐based system was utilized as an NIR PL‐type biosensor that detects Hg(II) through modification of flavin assembly on the SWNT surface.^[^ ^160^ ^]^ Moreover, flavin coating enhances thermal oxidative resistance of MWNT.^[^ ^161^ ^]^ In addition, a partially oxidized flavin‐coated MWNT was utilized to generate a hierarchical monoclinic MWNT‐nylon 6 nanocomposite that has high electrical conductivity (i.e., 100 S m^−1^).^[^ ^162^ ^]^ The facile chemical modification of flavins can be employed to flavin‐based SWNT and MWNT sorting and functionalization can be used in various optoelectronic fields.

Flavin has the advantage of having clear recognition depending on SWNT chirality and handedness due to the helical wrapping structure caused by hydrogen bonding between flavins as well as *π*–*π* interactions on the SWNT surface. However, there are relatively few studies on SWNT separation using flavin. Flavin, like DNA, is expected to show high single chirality separation efficiency when combined with separation methods such as IEC and ATPE. Flavin has the disadvantage of having a lower concentration of dispersed SWNTs compared to dispersants such as DOC, and it is difficult to visually discern the separated SWNTs due to the yellow color of flavin.

### 4.6 Gel Column Chromatography

Gel column chromatography (GCC) has been used to separate macromolecular biological substances such as proteins according to size and molecular weight. GCC is based on the principle of size‐exclusive chromatography in which separation of components of mixtures is accomplished by passing through a column containing porous gel beads. In this type of system, smaller particles more efficiently enter pores in gel beads than do larger particles so that particle separation is a consequence of different path lengths for elution through the column. Sephacryl S‐200HR gel is the most widely used in GCC sorting of SWNTs. The average pore size of Sephacryl S‐200 gel is 7.7 nm,^[^ ^163^ ^]^ which is smaller than the lengths of SWNTs, which are in the range of several hundred nanometers. As a result, it is difficult for SWNTs to diffuse into the pores.^[^ ^164^ ^]^ Thus, sorting of SWNTs is caused by differences in interactions between SWNTs and gel beads not by size and molecular weight.

**Figure 16:**
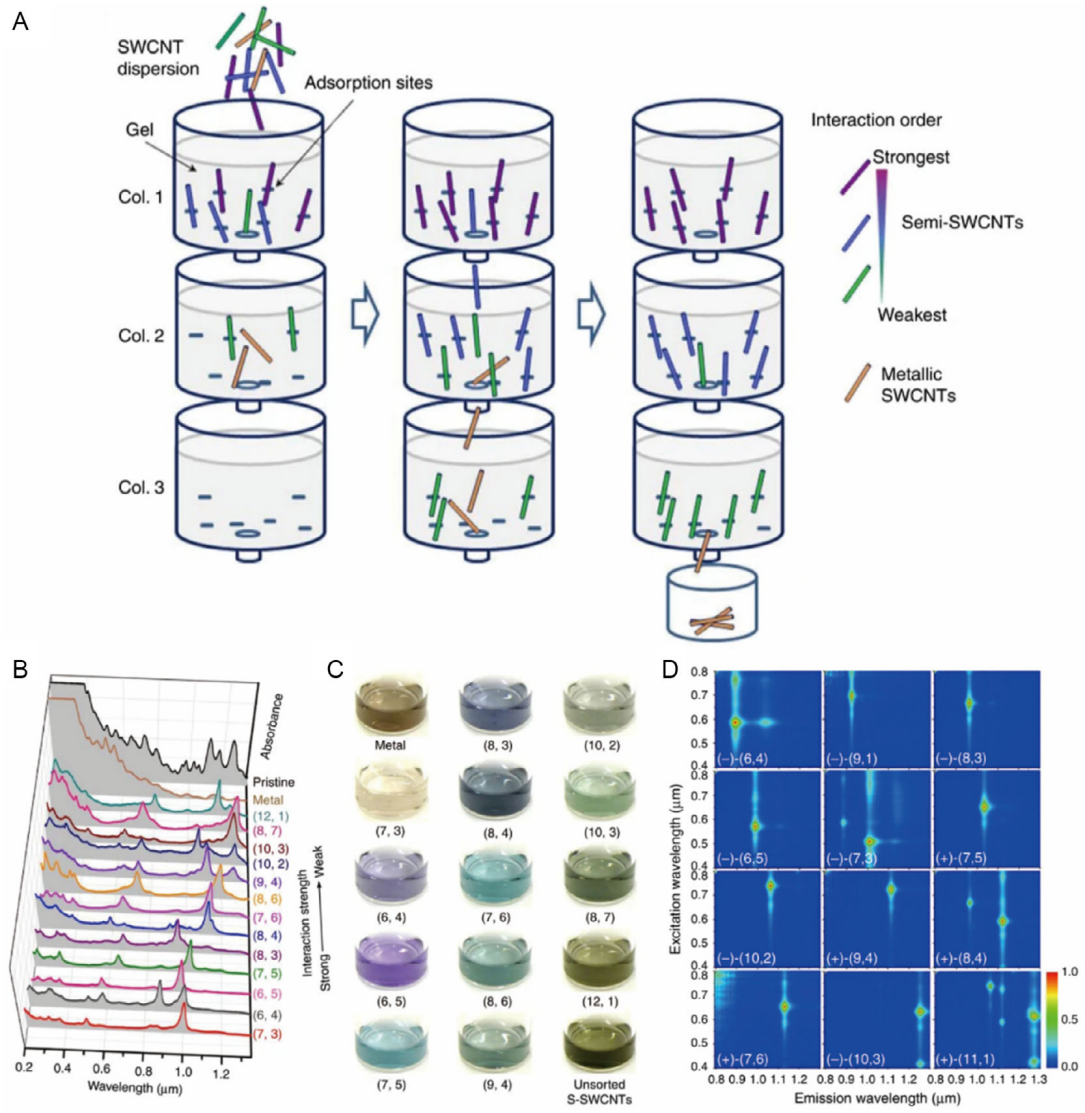
GCC‐based sorting of SWNTs according to type and handedness. A) Schematic of the single‐dispersant multicolumn gel chromatography process using SDS according to interaction between the (*n*, *m*) species and the gel. B) Absorption spectra and C) photographs of SWNTs sorted by type and electronic type. The spectra are ranked according to the strength of interactions between the (*n*, *m*) species and the gel. Reproduced with permission.^[^ ^130^ ^]^ Copyright 2011, Springer Nature. D) PLE maps of SWNTs sorted by handedness according to a stepwise elution with a mixed SDS/SC/DOC. Reproduced under terms of the CC‐BY license.^[^ ^168^ ^]^ Copyright 2016, Springer Nature.

In 2009, Tanaka and co‐workers^[^ ^165^ ^]^ studied the use of agarose gel, and in the same year Moshammer and co‐workers^[^ ^166^ ^]^ employed Sephacryl S‐200 gel for separation of SWNTs according to electronic type. Commonly, SDS/*s*‐SWNT interacts more strongly with both Sephacryl S‐200 and agarose gels than does SDS/*m*‐SWNT. This phenomenon is a consequence of the fact that *m*‐SWNT has a greater polarizability of positive charges induced by the anionic sulfate moiety in SDS. Therefore, interactions with the gel vary depending on the degree to which SWNTs are coated with a dispersant. In 2011, Liu and co‐workers^[^ ^130^ ^]^ developed single‐dispersant multicolumn gel chromatography using SDS and Sephacryl gel to isolate single‐chirality SWNT at the same time as *m*‐/*s*‐ separation takes place. As shown in **Figure** **16**A, in this method initially, SDS‐dispersed SWNTs are passed through the Sephacryl gel column several times so that they are effectively sorted in the order of their interactions with the gel. Next, isolated SWNTs are produced by elution using a high concentration of SDS (i.e., 5 wt%) so that adsorbed SWNTs are released. The overloading used in this method has a great effect on single‐type separation. For instance, when excessive amounts of the dispersed SWNT are loaded on the column, only SWNTs that have large interactions with the gel are more tightly held by gel. Thus, *m*‐SWNT, which is fully coated with SDS, does not bind tightly and is eluted first. Then, *s*‐SWNTs are sorted in an order corresponding to their bond curvature radius determined by *d*_t_ and *θ*. In Figure 16B, absorption spectra of the materials obtained using GCC are shown, with the sorted samples clearly distinguished according to color in Figure 16C.

In 2014, Liu and co‐workers^[^ ^167^ ^]^ succeeded in sorting SWNTs according to handedness using SDS, that arises from the small difference between the stereogenic centers in Sephacryl gel, a composite gel prepared by crosslinking of chiral allyl dextran with *N*,*N*′‐methylene bis(acrylamide). In a 2016 study by Wei and co‐workers,^[^ ^168^ ^]^ several *s*‐SWNTs were highly sorted according to handedness using mixed dispersants including SC and DOC. which possess multiple stereogenic centers. By varying the concentrations of mixed SC and DOC in the sorting process, differences in the organization ability of dispersants surrounding the SWNT are maximized due to their binding affinity differences. The purities of the sorted (*n*, *m*) species were confirmed by analysis of PLE maps (Figure 16D). Multiple follow‐up investigations have been carried out while extraction parameters^[^ ^169^, ^170^ ^]^ were varied and other dispersants were introduced for high purity.^[^ ^171^, ^172^ ^]^

These efforts demonstrated that GCC method has the characteristics of chromatography, so it has the advantage of being easy to scale up and continuous separation by controlling the amount of fractionation aliquot.^[^ ^173^ ^]^ Utilizing GCC, automated extraction systems via HPLC^[^ ^174^ ^]^ and the high purity of chirality extraction from high‐concentration dispersion^[^ ^175^ ^]^ are reported. Also, because it does not use another mobile phase including a dispersant with high binding force with SWNT for separation, GCC can obtain a dispersion with a high degree of individualization of SWNTs during the separation process. During continuous separation, there is an advantage that the binding force can be easily adjusted by adjusting the temperature, pH, and type and concentration of the dispersant at each step. Additionally, there is an advantage that there are no additives remaining after separation other than the dispersant and SWNT. However, the disadvantage is that multiple steps of separation must be performed to separate single chirality. In addition, before proceeding with GCC, since the pore size of the gel is smaller than the length of the SWNTs, the SWNTs may be adsorbed on the gel surface and be lost. Therefore, for effective separation, the length of the SWNTs must be reduced by ultrasonic pulverization for an extended period of time.^[^ ^176^, ^177^ ^]^ Even though the SWNT length has been reduced, reuse of the gel is limited due to irreversibly adsorbed SWNTs, which also remains a cost limitation.^[^ ^178^ ^]^

### 4.7 Aqueous Two‐Phase Extraction

ATPE is a method that can be employed to separate not only cell particles, DNA, and proteins but also microorganisms and viruses. Phase separation occurs from an aqueous solution above a critical concentration of two water‐soluble polymers with different hydrophilicities (*i.e.*, hydrophilic dextran (DEX) and hydrophobicity poly(ethylene oxide) (PEG)).^[^ ^179^ ^]^ Therefore, phase partitioning of two polymers enables extraction of a species from one phase into another.

**Figure 17:**
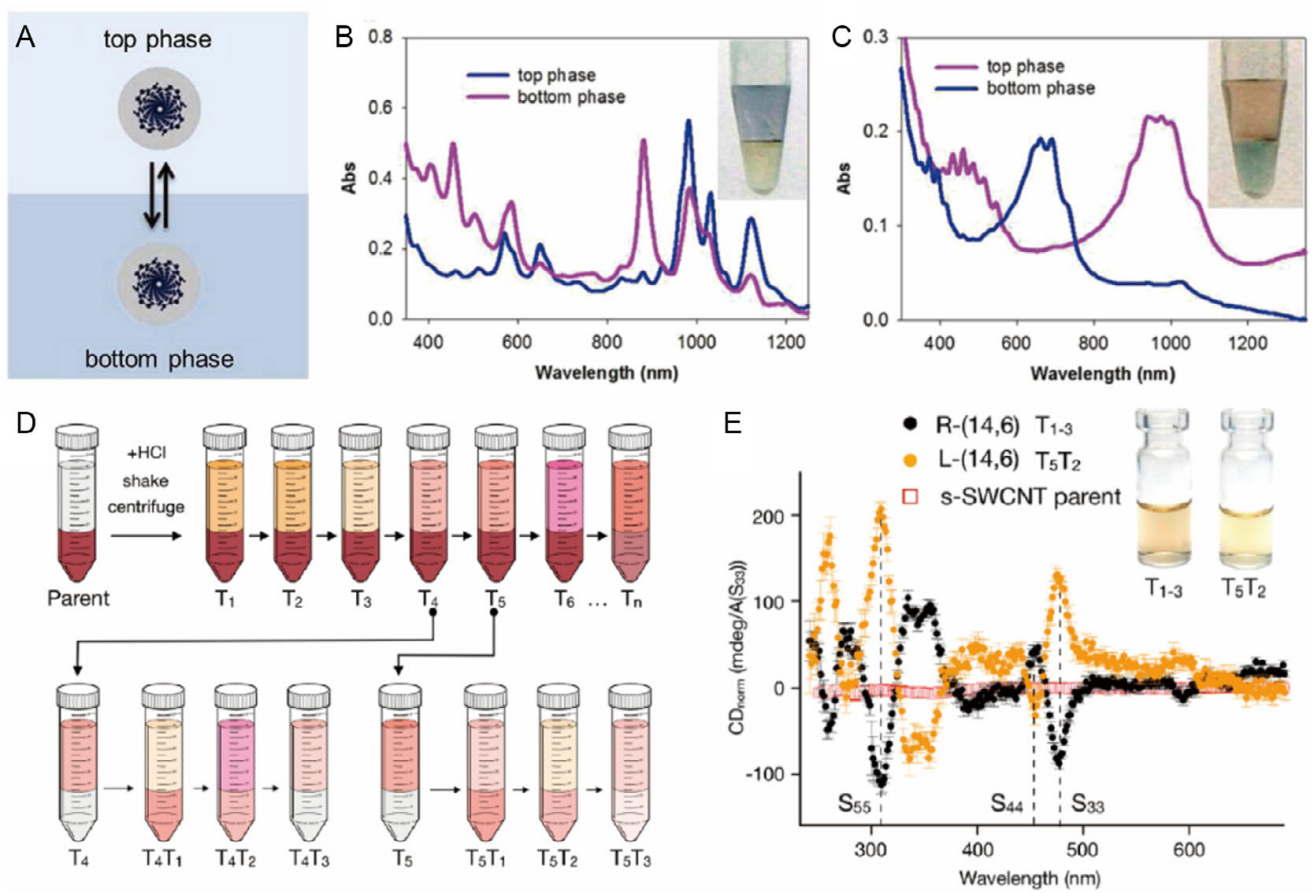
ATPE‐based sorting of SWNT according to *d*_t_, electronic type, and handedness. A) Schematics of the two‐phase system. Top and bottom phases are PEG‐rich and DEX‐rich, respectively. B) Partition of small *d*_t_ SWNT. C) Partition of large *d*_t_ arc‐discharge tubes. UV–vis–NIR absorption spectra of the top (pink) and bottom (blue) phases are shown. Inset in each panel shows a photograph of the partitioned sample. Reproduced with permission.^[^ ^180^ ^]^ Copyright 2013, American Chemical Society. D) Illustration of the use of repetitive ATPE of the SWNT suspension for enantiomer sorting. E) CD spectra of the two (14,6) enantiomers. Reproduced under terms of the CC‐BY license.^[^ ^181^ ^]^ Copyright 2020, American Chemical Society.

Sorting SWNTs using the ATPE method was first reported by Khripin and coworkers^[^ ^180^ ^]^ in 2013. In the study, DEX and PEG were mixed to create a phase‐separated aqueous mixture (**Figure** **17**A), to which SDS was added to a SWNT dispersed in the SC to finely tune the hydrophilicity of the dispersant wrapping. As a result, SWNT spontaneously partitions between the PEG‐ or DEX‐rich phases according to its *d*_t_ and electronic type (Figure 17B,C, respectively). Relative hydrophobicities of electronic types and *d*_t_ (*i.e.*, greater hydrophobicity: *s*‐SWNT, larger *d*_t_ SWNT) are assigned as the basis for the extraction order.

The ATPE‐based SWNT sorting method, proposed by Khripin and coworkers,^[^ ^180^ ^]^ has been continuously developed and utilized for sorting SWNTs according to handedness^[^ ^181^, ^182^, ^183^ ^]^ as well as electronic properties^[^ ^180^, ^184^, ^185^, ^186^ ^]^ and types.^[^ ^129^, ^180^, ^185^, ^187^, ^188^, ^189^ ^]^ Li and co‐workers^[^ ^181^ ^]^ described sorting of enantiomers of SWNTs that have *d*_t_ values larger than 1.1 nm using a two‐step ATPE technique (Figure 17D). Competitive adsorption of dispersants to the SWNTs governs partitioning in the ATPE separation process, and inclusion of acids enables fine control of the SWNT sorting by doping. In the sorting process, concentrations of the three dispersants, SC, DOC, and SDS, were used to sort SWNT according to their properties, especially *d*_t_ values and handedness (Figure 17E).

This ATPE sorting method can be easy scaled‐up,^[^ ^190^ ^]^ and it has the advantage of being fine‐tunable through the control of intrinsic and extrinsic sorting factors, which will be described below. In addition, among the various methods presented so far, ATPE has succeeded in separating the single chirality of multiple species and has also succeeded in separating the chirality of multiple species of *m*‐SWNT.^[^ ^185^ ^]^ In addition, single‐chirality separation has been achieved with just one extraction step by introducing polymers^[^ ^191^ ^]^ and nonionic dispersants.^[^ ^192^ ^]^ Furthermore, a new separation technique^[^ ^193^ ^]^ has been recently proposed, achieving single chirality separation by extracting the single chirality, initially extracted by the water system, into an organic system with PFO dispersant. However, its disadvantage is that it is a repetitive and continuous separation process whose the concentration of the medium polymer and the dispersant must be carefully controlled.^[^ ^194^ ^]^ Additionally, if the individualization of SWNTs is not done well, the bundle forms a film at the ATPE interface, causing loss, which has the limitation of impeding future separation. Therefore, this method requires the use of multiple presampling processes including rate‐zonal centrifugation using DGU to remove SWNT bundles prior to sorting involving tedious repetitive extractions. Despite this pretreatment process, the SWNT bundle formed by the medium environment that changes as it passes through the interface remains a limitation. In addition, since the widely used DOC/SDS dispersant is used, it is difficult to separate SWNTs with a diameter larger than 1.8 nm.^[^ ^190^ ^]^ Moreover, complete removal of polymers and other additives such as PEG or DEX used in the sorting process could be difficult to accomplish.^[^ ^194^ ^]^

### 4.8 Other Sorting Methods

The sorting methods discussed above accomplish targeted separation of SWNT species by introducing various types of dispersants. To improve SWNT purities, methods have been devised that take advantage of unique properties of synthesized dispersant derivatives that make liberation of pure SWNTs possible. For example, PFO‐based dispersant derivatives have been developed to selectively disperse *s*‐SWNTs in organic solvents by taking advantage of variations in solubility according to electronic type,^[^ ^146^ ^]^ and the modified dispersants have been developed as dispersing agents for SWNTs that can be effectively removed by adjusting pH.^[^ ^195^ ^]^

Methods for isolation of pure SWNTs without using dispersants include electrical and chemical treatment, in which SWNTs are selectively destroyed due to differences in their bandgap structures or electrical properties and their consequent responses to various external stimuli (e.g., electricity, light, plasma, and microwave). Selective separation of SWNTs using electric currents is based on Joule heating, which occurs in highly electrically conductive materials like *m*‐SWNTs. In 2014, Otsuka and co‐workers^[^ ^196^ ^]^ developed a procedure to isolate *m*‐SWNTs using Joule heating. The method involves growing SWNTs in a horizontal orientation and then coating them with an organic film. When an electric current is applied, only the film on *m*‐SWNTs melts as a consequence of Joule heating, which enables isolation of pure s‐SWNT. In 2008, Zhang and co‐workers^[^ ^197^ ^]^ utilized a strong xenon lamp to selectively remove *m*‐SWNTs with an average *d*_t_ of 1.2 nm, which absorb light in the visible region. In addition, a monochromatic laser was utilized for selectively removing *s*‐SWNTs.^[^ ^198^ ^]^ Methane^[^ ^199^ ^]^ and hydrogen^[^ ^200^ ^]^ plasma not only selectively destroy *m*‐SWNTs, they also vary the etching rate of SWNT according to *d*_t_ and *θ* as the annealing temperature is increased. The use of microwaves in a separation method is a result of the fact that *m*‐SWNTs have a band structure continuum in the microwave region. Consequently, selective absorption and destruction of *m*‐SWNTs can be induced by irradiation with 2.45 GHz microwaves.^[^ ^201^ ^]^ Finally, different electronic‐type SWNTs can be separated using chemical methods. For example, covalently bonding a diazonium derivative to SWNT enables electron transfer to occur from the SWNT to diazonium center in an SWNT electronic‐type and bandgap structure‐dependent manner.^[^ ^202^ ^]^ Although the above separation methods are capable of separating SWNTs according to electronic types on a large scale, they do not achieve complete sorting based on single chirality and handedness. In any event, fabrication of high‐integration SWNT‐based transistors is simplified utilizing these separation methods.

### 4.9 Comparison of Dispersants Type for Each Method

Earlier, we described various separation methods that have been employed to sort SWNTs according to electronic type, chirality, and handedness. These methods take advantage of interactions between dispersants and SWNTs. Thus, comparisons of separation methods that utilize various dispersant types and concentrations would be helped by an understanding of the sorting schemes given in **Table** **2**. First, schemes that use nonclassical dispersants like DNA, PFO, and porphyrins normally utilize relatively lower concentrations of dispersants (i.e., ≤0.5 w./v%). The reason for this is that preferential interactions take place between the sidewalls of SWNTs and specific functional groups of those dispersants (e.g., nucleobase, *π*‐conjugated fluorene, and porphyrin ring). In addition, the binding affinities between dispersants and SWNT have been changed by modifying the polymer structure, such as changing the DNA sequence, side chain length and additional aromatic units in PFO, and the intermediate bridge structure of the porphyrin tweezers. These structural changes enable distinction between types of SWNTs using only one dispersant by finely controlling the helical pitch, polymer backbone rigidity, torsional angle, etc. Although flavins belong to the molecular dispersant group, their separation of SWNTs is accomplished at low concentrations of around 0.1 w./v%. Also, due to the unique supramolecular helix structure of flavins on SWNT surfaces, *s*‐SWNT (8,6) chirality and *M‐*SWNT, both of which bind strongly to flavins, can be selectively separated.

**Table 2:** Various dispersant systems fused in methods for sorting SWNTs by type

System	Sorting type	Dispersant(s)	Concentration [×10^−2^ w./v%]	Note	Year	Ref.
DNA	Electronic type	C,T‐dominant ssDNA	10	IEC	2003	[113]
	Chirality	G,T‐dominant ssDNA	50	IEC	2007	[233]
		ssDNA	10	IEC	2009	[114]
		ssDNA	20	ATPE	2014	[188]
		A,T,C‐dominant ssDNA	20–25	ATPE	2019	[234]
	Handedness	ssDNA	10–20	ATPE	2016	[115]
DGU	Electronic type	SDS/SC	40/160	–	2006	[124]
	Chirality	G,T‐dominant ssDNA	10	–	2005	[122]
		SC	200	–	2006	[124]
	Handedness	SC	100	–	2009	[127]
		SDS/SC	17.5/70	–	2010	[121]
PFOs	Electronic type	P(C12‐FO*‐co*‐Cz)a), P(C16‐FO*‐co*‐DMB)a)	4 or 6	–	2021	[235]
	Chirality	P(C8‐FO), P(C12‐FO), P(C8‐FO*‐co*‐BTH)a)	1.4	–	2009	[236]
		P(C6‐FO), P(C8‐FO), P(C8‐FO*‐co*‐Ph)a), P(C8‐FO*‐co*‐BTH)	6	–	2007	[132]
		P(C10‐FO)‐*block*‐P(MB‐FO)a)	1.7	–	2011	[237]
		P(C12‐FO), P(C8‐FO*‐co*‐BTH)	6.7	–	2011	[238]
		P(C8‐FO), P(C6‐FO*‐co*‐Ant)a), P(C8‐FO*‐co*‐BTH)	6.3–31.3	–	2007	[133]
	Handedness	P(C8‐FO)‐*block*‐P((*R*)‐BINAP)a)or P(C8‐FO)‐*block*‐P((*S*)‐BINAP)	10	–	2012	[134]
		P(C12,C2‐FO‐*N*)	3	–	2021	[135]
Porphy‐rins	Handedness	Por‐(*m*‐Ph)‐Porb)	5.5	–	2007	[50]
		Por‐Py‐Porb)	2.5	–	2007	[53]
		Por‐PHE‐Porb)	2.5	–	2010	[151]
		Por‐(3,6‐Cz)‐Por	3.3	–	2011	[152]
Flavins	Electronic type	FC12	10	–	2015	[154]
		FC12	2.510	*s*‐SWNT *m*‐SWNT	2016	[153]
		Alkyl flavin derivatives	10	–	2020	[157]
	Chirality	FMN	10	–	2008	[65]
		FC12	10	**–**	2018	[239]
	Handedness	FMN	10	**–**	2012	[155]
		FMN	2.5–510–20	*M*‐*M*‐ & *P*‐	2017	[210]
GCC	Electronic type	SDS/SC	100/100	–	2009	[166]
		SDS	100	pH	2013	[223]
		SDS/DOC	100/100	Temp.	2015	[215]
		Triton X‐405/Brij L23	280/100	–	2016	[205]
		SDS/DOC	50/200	Temp.	2020	[216]
	Chirality	SDS	200	–	2011	[130]
		SDS	200	Temp.	2013	[213]
		SDS/Bile salt	200/30–70	–	2015	[220]
		SDS/SC/DOC	100/50/6–20	Temp.	2021	[217]
	Handedness	SDS/SC/DOC	50/50/1–7	–	2016	[168]
		SDS/SC/DOC	50/50/2.7–3.2	–	2017	[211]
ATPE	Electronic type	SDS/SC	70/90	–	2013	[180]
		SDS/DOC	60/6	pH	2019	[224]
		SDS/SC/DOC	70/90/2	–	2020	[181]
	Chirality	SDS/DOC	25–170/2.3	–	2014	[129]
		SDS/SC/DOC	97/42/5	–	2014	[189]
		SDS/SC	100/90	–	2015	[186]
		DOC	8.8	–	2015	[187]
		SDS/SC	20–180/4.5	–	2015	[185]
		SDS/SC	70/90	–	2017	[184]
	Handedness	SDS/DOC	10–70/2	–	2014	[182]
		SDS/SC/DOC	22.5/90/15	pH	2020	[181]

a) P; Poly, C_n_‐FO; di‐*n*‐alkyl fluorene (*n* = number of carbons in *n*‐alkyl side chain), Cz; carbazole, DMB; dimethoxybenzene, BTH; benzothiadiazole, Ph; phenylene, MB; (*S*)‐(+)‐2‐methylbutyl, Ant; anthracene, BINAP; binaphthol.

b) Por; porphyrin, Py; pyridine, PHE; phenanthrene.

Separation methods that utilize DGU, GCC. and ATPE are performed for the most part using commonly used molecular dispersants. Most of these separation methods employ dispersant concentrations of 0.4 w./v% or higher in order to form dispersant micelles on the SWNT surface. In addition, recent methods using molecular dispersant have employed mixed‐dispersant systems (as indicated by asterisk symbol in the Table 2) to increase separation efficiencies. Unlike single dispersant‐based sorting, mixed dispersants have additional binding properties that can be used to precisely manipulate *K*_a_ between dispersants and SWNTs. As shown in the legend of Table 2, some methods for sorting SWNT rely on pH, temperature, as well as the dispersant concentration changes. In next section, we will explain how intrinsic and extrinsic parameters affect the *K*_a_ for equilibria between dispersants and SWNTs.

## 5 Parameters That Control Binding Affinity

SWNT sorting using several methods discussed above is based on small differences in the interactions of dispersants with different SWNT species. As a result, it is important to understand how these differences depend on the supramolecular structure of a dispersant and the manner in which it is wrapped around the SWNT. Self‐assembly of dispersants and SWNTs is a dynamic chemical equilibrium process that can be governed by both externally adjustable factors (extrinsic parameters) and indigenous properties of the dispersant (intrinsic parameters). For example, the extent to which dispersants aggregate in solution depends on extrinsic factors such as on the temperature^[^ ^203^, ^204^ ^]^ and pH,^[^ ^205^, ^206^ ^]^ which have an effect on properties including critical micelle concentration and *γ*. In contrast, structural features of the dispersant and the SWNT, including *d*_t_, bandgap, electronic type, and handedness, are intrinsic parameters that affect the equilibrium process. The equilibrium constant for the wrapping process between the dispersant and SWNT, referred to as binding affinity, is governed by both parameters. As a result, an understanding of the extrinsic and intrinsic parameters should provide the foundation for designing new sorting materials and methods.

### 5.1 Intrinsic Parameters (i.e., Bandgap, Electronic Type, and Handedness)

The supramolecular structure of the dispersant surrounding the SWNT is modified via altering the equilibrium reaction of dispersant self‐assembly. The temperature, concentration, and pH are suggested to control this chemical equilibrium reaction. Compared to extrinsic parameters that change the configuration or bonding strength of the dispersant assembly, intrinsic parameters that govern binding affinities between dispersants and SWNTs include the physical properties of SWNTs such as bandgap, electronic type, and handedness.

#### 5.1.1 Bandgap and Electronic Type

In 2003, Krupke and co‐workers^[^ ^207^ ^]^ developed a method to sort *m*‐SWNT from a SWNT suspension that uses alternating current (AC) dielectrophoresis. In this protocol, a drop of an SWNT dispersion is applied to the chip which is then placed in a microelectrode array generated electric field. Due to the polarizability difference between the two species with respect to a solvent, *m*‐ and *s*‐SWNT move in opposite directions in the electric field gradient. During dielectrophoresis, *m*‐SWNT is attracted to a pair of microelectrodes, which has an alternating current field gradient whereas *s*‐SWNT is expelled. Proof of the effectiveness of sorting is given by a comparative Raman spectroscopy study on the dielectrophoretically deposited SWNT and on a reference sample.

**Figure 18:**
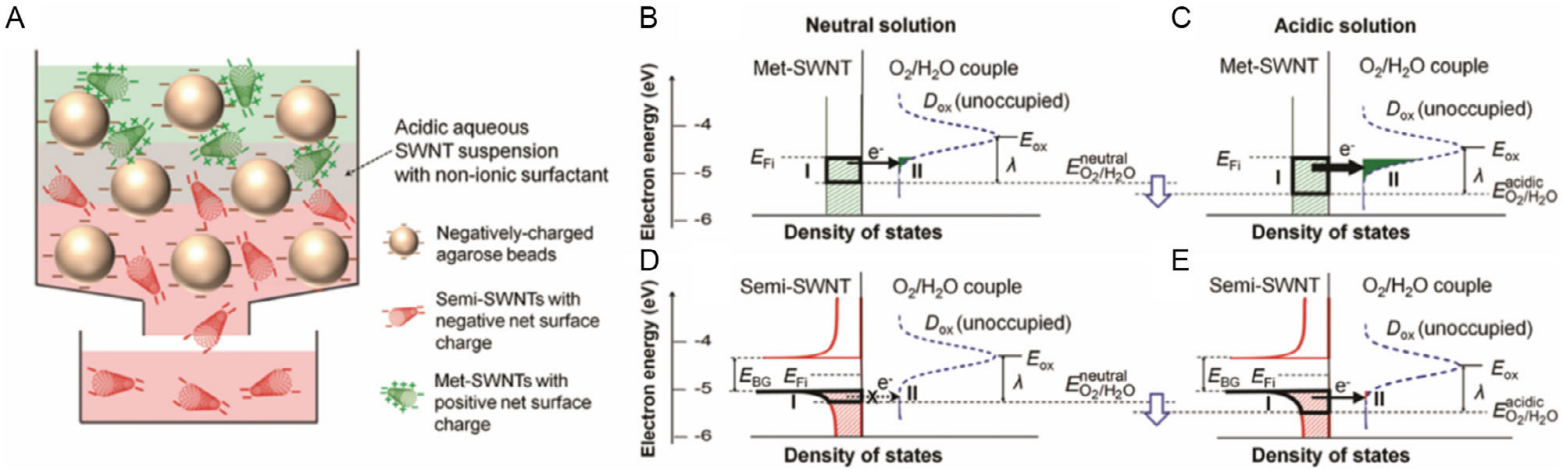
Differences in electron transfer according to electronic type. A) Schematic of the CSR chromatographic sorting process. B–E) Illustration of the MG theory for differential charging of *m*‐ versus *s*‐SWNTs by bandgap‐dependent heterogeneous electron transfer from SWNTs to O_2_/H_2_O couple. Electron transfer from *m*‐SWNT to O_2_/H_2_O couple in B) neutral and C) acidic solution. Electron transfer from *s*‐SWNT to O_2_/H_2_O couple in D) neutral and E) acidic solution. Reproduced under terms of the CC‐BY license.^[^ ^205^ ^]^ Copyright 2016, American Chemical Society.

Various sorting methods such as electrical breakdown^[^ ^201^, ^208^, ^209^ ^]^ directly exploit intrinsic differences in SWNT band structures to achieve highly effective separation of *m*‐SWNT. However, these methods are not readily scalable and not highly selectivity. Also, known bulk sorting techniques such as GCC, DGU, ATPE, and electrophoresis are less sensitive because they are based on subtle differences between SWNTs and dispersants due to differences in SWNT polarizability. In 2016, Wang and co‐workers^[^ ^205^ ^]^ designed a novel synergistic chromatographic sorting system based on charge sign reversal (CSR) that directly exploits differences in SWNT band structure (**Figure** **18**A). This method was used for large‐scale separation of ultrahigh‐purity SWNT by electronic type. The synergistic chromatographic system utilizes SWNTs dispersed with a nonionic dispersant, which minimizes Coulomb interaction between the SWNT and negatively charged hydrophilic agarose beads. By lowering the pH of the medium, the resulting positively charged SWNT encased by the nonionic dispersant results in better separation according to electronic types. pH modulation induces redox reactions using an O_2_/H_2_O redox couple (2 H^+^ + 1/2O_2_ ⇌H_2_O) at an optimized pH to generate contrasted charge surface for *s*‐ and *m*‐SWNT. The heterogeneous electron transfer from a SWNT to the O_2_/H_2_O couple lowers the Fermi level, resulting in a difference in reaction rate according to the Marcus–Gerischer (MG) theory. As shown in Figure 18B,C, the VB of *m*‐SWNTs is within the reorganized energy (*λ*), facilitating electron transfer from *m*‐SWNT to O_2_/H_2_O redox couple, whereas that of *s*‐SWNT is about energy of *λ* and restricted electron transfer (Figure 18D,E). Those electron transfer reaction is dependent on redox depth of the O_2_/H_2_O couple. Unlike *s*‐SWNT which was eluted first, the *m*‐SWNT remained in the column due to electrostatic interaction with negatively charged beads. This method achieved 99.94% of *s*‐SWNT purity using just one column chromatography purification. This facile CSR‐based sorting technique is robust and enables to sort SWNT with a broad *d*_t_ range.

#### 5.1.2 Handedness

**Figure 19:**
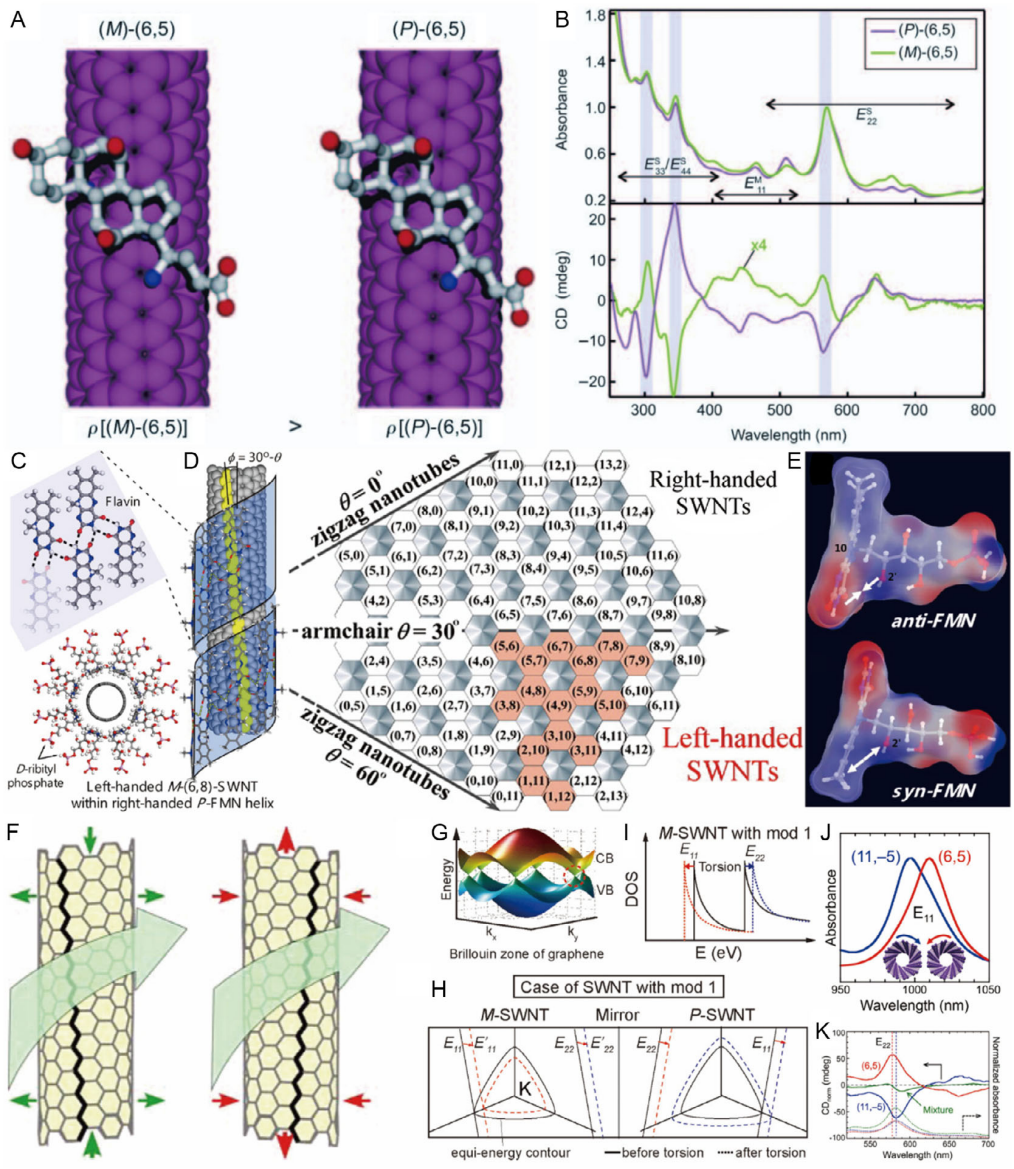
Sorting handedness of SWNT. A) Schematic of the interaction of *M‐* and *P‐*(6,5) SWNTs with SC, which causes small differences in the *ρ* of SWNT enantiomers. B) Absorption and CD spectra of *P‐*(6,5) SWNTs and *M‐*(6,5) SWNTs. Reproduced under terms of the CC‐BY license.^[^ ^127^ ^]^ Copyright 2009, Springer Nature. C–E) Adjacent hydrogen bonds stabilize the flavin helical ribbon C) wrapped around SWNTs D) with the *d*‐ribityl phosphate side groups providing solubility in water. E) Two conformations of *R*‐FMN. (F) The right‐handed FMN helix exerts torsional strain on the nanotube that, in the case of left‐handed chiral SWNTs, causes axial compression and *d*_t_ dilation (left) as opposed to the case of right‐handed SWNT in which it creates axial elongation and *d*_t_ contraction (right). Reproduced with permission.^[^ ^155^ ^]^ Copyright 2012, American Chemical Society. G–I) Modality‐ and handedness‐dependent *e*_*ii*_ changes of SWNTs in the Brillouin zone. G) Energy dispersion according to reciprocal lattice *k*_1_ and *k*_2_.H) Optical transition changes of (left) *M‐*SWNT and (right) *P‐*SWNT according to unidirectional torsion (red arrows). I) Resulting optical transition shifts of *M‐*SWNT with mod 1. Reproduced with permission.^[^ ^210^ ^]^ Copyright 2017, American Chemical Society. J) Absorption spectra of (6,5) and (11,–5) at different wavelengths due to handedness‐dependent interactions with the FMN molecule. K) CD spectra of the *e* ^S^_22_ region accompanied by absorption spectra of (6,5) and (11,–5). Reproduced with permission.^[^ ^211^ ^]^ Copyright 2017, American Chemical Society.

In methods for separation of SWNTs according to handedness, the single enantiomers of chiral dispersants interact differently with *M‐* and *P‐*SWNT, as described above in discussions of methods based on diporphyrin nanotweezer^[^ ^50^, ^53^, ^151^, ^152^ ^]^ and PFO.^[^ ^134^ ^]^ In a study of another method for this purpose, in 2009, Green and co‐workers^[^ ^127^ ^]^ sorted enantiomers of SWNTs using DGU and the dispersant SC. Since SC is chiral (**Figure** **19**A) a slight energy difference exists in its interactions SWNTs having different handedness, which causes the complexes to have different *ρ*, making it possible to sort them using DGU. In contrast, in GCC, SWNT handedness can also be sorted using achiral SDS.^[^ ^167^ ^]^ In this case, the existence of a stationary phase comprising one enantiomer of chiral Sephacryl gel enables sorting since the SWNTs having different handedness cannot be distinguished using absorption spectroscopy and PLE mapping. They can be distinguished using CD (Figure 19B).^[^ ^121^, ^168^ ^]^

In the flavin system, the wavelength shift of the peak at absorption and PL induced by helical flavin wrapping is even larger according to handedness.^[^ ^155^ ^]^ As shown in Figure 19C,D, FMN helically surrounds the SWNT through hydrogen bonding interactions and its *d*‐ribityl phosphate side group gives it solubility in water. *P*‐FMN exists in two conformations (Figure 19E). Electrostatic attraction between the uracil moiety and 2′‐OH on the ribose side chain stabilizes the anticonformer, which leads to the *P‐*FMN helical structure. *P‐*FMN selectively wraps *M‐*SWNT (Figure 19F). When *P‐*FMN wrapping occurs *P‐*SWNT is stretched in the axial direction and contracted in the radial direction. On the contrary, when wrapped by *P*‐FMN, *M*‐SWNT is relaxed in the radial direction so that interaction between the SWNT surface and the FMN increases, leading to energetic stabilization and preferential binding.

The peak shift occurring between the SWNTs with different handedness upon *P*‐FMN assembly can be explained by a wave vector shift taking place near the K point of Brillouin zone of the graphene reciprocal lattice in which VB and CB are in contact (Figure 19G).^[^ ^210^ ^]^ In Figure 19H, the energy dispersion contour maps near the K points of *M‐*SWNT and *P‐*SWNT which are mirror images is given. Due to tight binding of the FMN helix, *M‐*SWNT with mod = 1 undergoes a redshift in *e* ^S^_11_ and blueshift in *e* ^S^_22_ because of unidirectional strain caused by the chiral dispersant. Conversely, *P‐*SWNT undergoes *e* ^S^_11_ blueshift and *e* ^S^_22_ redshift. The energy shifts can be plotted in terms of DOS (Figure 19I). These results can be confirmed by analysis of experimental absorption and PL data. In the FMN system, the absorption wavelength shift of handed SWNTs by FMN is observed in Figure 19J. That the SWNTs have been sorted according to handedness can be verified by comparing their CD and absorption spectra (Figure 19K).^[^ ^211^ ^]^ This method shows that a difference in the force applied to the SWNT enantiomers is a consequence of the manner by which FMN wraps and organizes the SWNT surface. This concept could be the basis for the discovery of dispersants that form arrays around SWNTs for handedness separation.

### 5.2 Extrinsic Parameters (i.e., Temperature, Dispersant Concentration, pH, and Aging Time)

#### 5.2.1 Temperature

**Figure 20:**
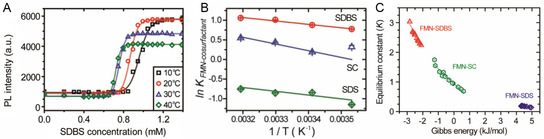
Temperature effect on SDS, SC, and SDBS replacement of FMN‐wrapped SWNT A) PL intensity traces of SDBS replacement from FMN‐wrapped (6,5) SWNT at different temperatures. B) Plots of the natural log of equilibrium constants *K* versus 1/T for SDS, SC, and SDBS replacement of FMN‐wrapped (6,5) SWNT at different temperatures. C) Plot of the equilibrium constant *K* versus Gibbs energy changes according to the exchange dispersant. Reproduced with permission.^[^ ^75^ ^]^ Copyright 2013, American Chemical Society.

The process involving formation of an assembly between a dispersant and SWNTs is governed by changes in enthalpies and entropies that are associated with binding energies and disorder. The effect of temperature on these energy changes has been evaluated in several thermodynamic studies.^[^ ^75^, ^103^, ^212^ ^]^ Interactions between the dispersants and SWNTs and dispersant itself, including vdW or hydrogen bonding, are noncovalent in nature and, as a result, they are one order of magnitude smaller than those involved in covalent bonding. Equilibrium constants associated with reversible processes governed by these weak interactions are modulated by varying the temperature. In 2013, Oh and co‐workers^[^ ^75^ ^]^ carried out a thermodynamic analysis of *K*_a_ values for equilibria between FMN‐wrapped and various dispersant‐wrapped SWNTs. The TP traces given in **Figure** **20**A show that the equilibrium constant for SDBS displacement of FMN‐wrapped (6,5) SWNT increases with increasing temperature from 10 to 40 °C. Thus, the [FMN]/*K* corresponding to the relative binding affinity value decreases. The 1/T versus log K plots in Figure 20B show that *K* changes with temperature follow the same trends when SDBS, SC, and SDS are employed as dispersants. Analysis of the enthalpy, entropy, and Gibbs free energy changes that correspond to extrinsic and intrinsic parameters can be calculated using the van't Hoff equation. The results (Figure 2C) show that Gibbs free energy changes are dependent on SWNT type.

**Figure 21:**
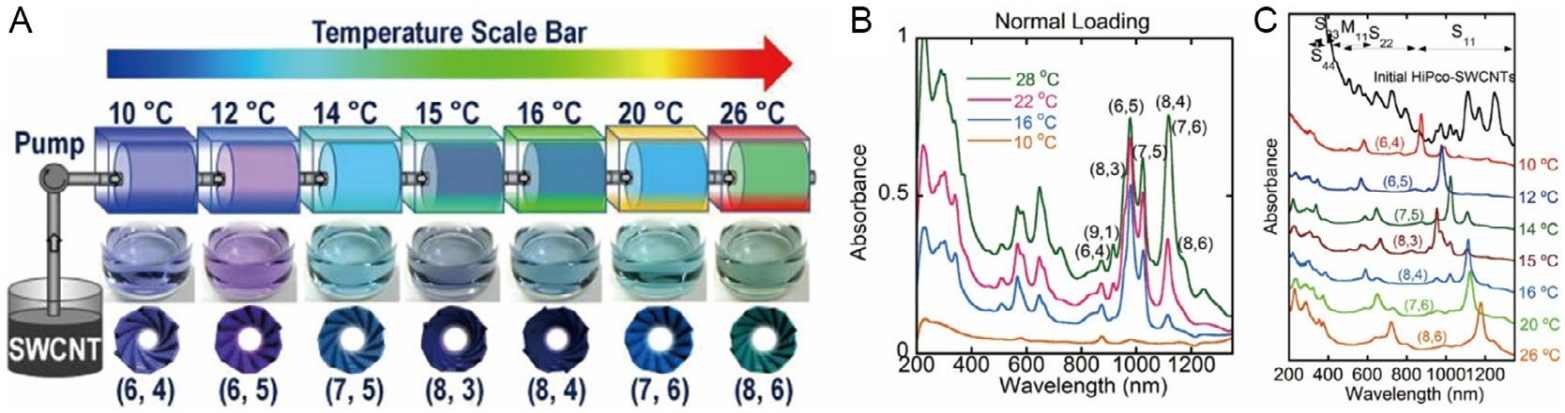
Temperature effect on sorting SWNT using GCC. A) Schematics of a multistage temperature‐controlled GCC system using SDS. B) Absorption spectra of the sorted SDS‐SWNT dispersion loaded on a column with different temperature conditions. C) Absorption spectra of sorted SWNT by *d*_t_. Reproduced with permission.^[^ ^213^ ^]^ Copyright 2013, American Chemical Society.

Attempts have been made to sort SWNT species by controlling the temperature during separation processes such as DGU,^[^ ^120^ ^]^ GCC,^[^ ^213^ ^]^ and ATPE.^[^ ^187^, ^189^, ^214^ ^]^ Among these approaches, the GCC method enabled individualization of SWNTs on the basis of temperature‐dependent *K*_a_ changes. In 2013, Liu and co‐workers^[^ ^213^ ^]^ reported that an improved sorting efficiency of SWNT type can be accomplished using SDS as the dispersant and temperature‐controlled GCC. In this procedure (**Figure** **21**A), elution is repeatedly performed on the SDS‐containing SWNT mixture loaded on the gel while the temperature is increased to as low as 10 °C. In GCC separations, the temperature dependence of interactions between SWNTs and gels serves as the basis for sorting (Figure 21B). It was suggested that as the temperature is lowered, the solubility of SDS in the dispersion is lowered, and the SDS coating surrounding the SWNT is strengthened, which lead to weakening of the interaction of the SWNT with the gel (Figure 21C). This observation is consistent with the trend observed in a study conducted by Oh and co‐workers,^[^ ^75^ ^]^ which showed the FMN *K*_a_ increases with decreasing temperature.

In 2015, Yahya and co‐workers^[^ ^215^ ^]^ reported that the inverse correlations existing between the purities of adsorbed *s*‐SWNT and eluted *m*‐SWNT depend on temperature. As the temperature is increased, the binding affinity between *s*‐SWNT and SDS increases leading to a greater amount of *s*‐SWNTs adsorbed on the gel. In 2020, Yoo and co‐workers^[^ ^216^ ^]^ reported that the ratio of *m*‐/*s*‐SWNT adsorbed to the gel increases as the temperature is increased. Removal of the dissolved gases such as molecular oxygen leads to dedoping the SWNT. As a result of redox coupling, the number of SDS surrounding reduced *m*‐SWNT is lowered according to charge balance, and interaction of the *m*‐SWNT with the gel therefore increases. In a recent 2021 study, Yang and co‐workers^[^ ^217^ ^]^ successfully sorted SWNTs in the zigzag and near‐zigzag regions with *θ* smaller than 20°. Although separation of these SWNTs is known to be difficult, this effort demonstrated that high‐purity SWNTs can be generated using temperature‐controlled GCC with a SDS/SC mixed‐dispersant system.^[^ ^217^ ^]^

#### 5.2.2 Dispersant Concentration

When a dispersant binds to a SWNT, a supramolecular assembly via noncovalent interaction is formed on the SWNT surface in a dynamic equilibrium involving reversible adsorption and desorption.^[^ ^218^ ^]^ As its concentration increases, the dispersant becomes more densely adsorbed to the SWNT according to the Le Chatelier principle. Since the dispersant both individualizes and disperses SWNTs, along with carbonaceous impurities and bundles, selecting an optimal dispersant concentration according to dispersant type is important.^[^ ^219^ ^]^ Also, the degree of dispersion depends on the SWNTs species and the SWNT/dispersant ratio.

**Figure 22:**
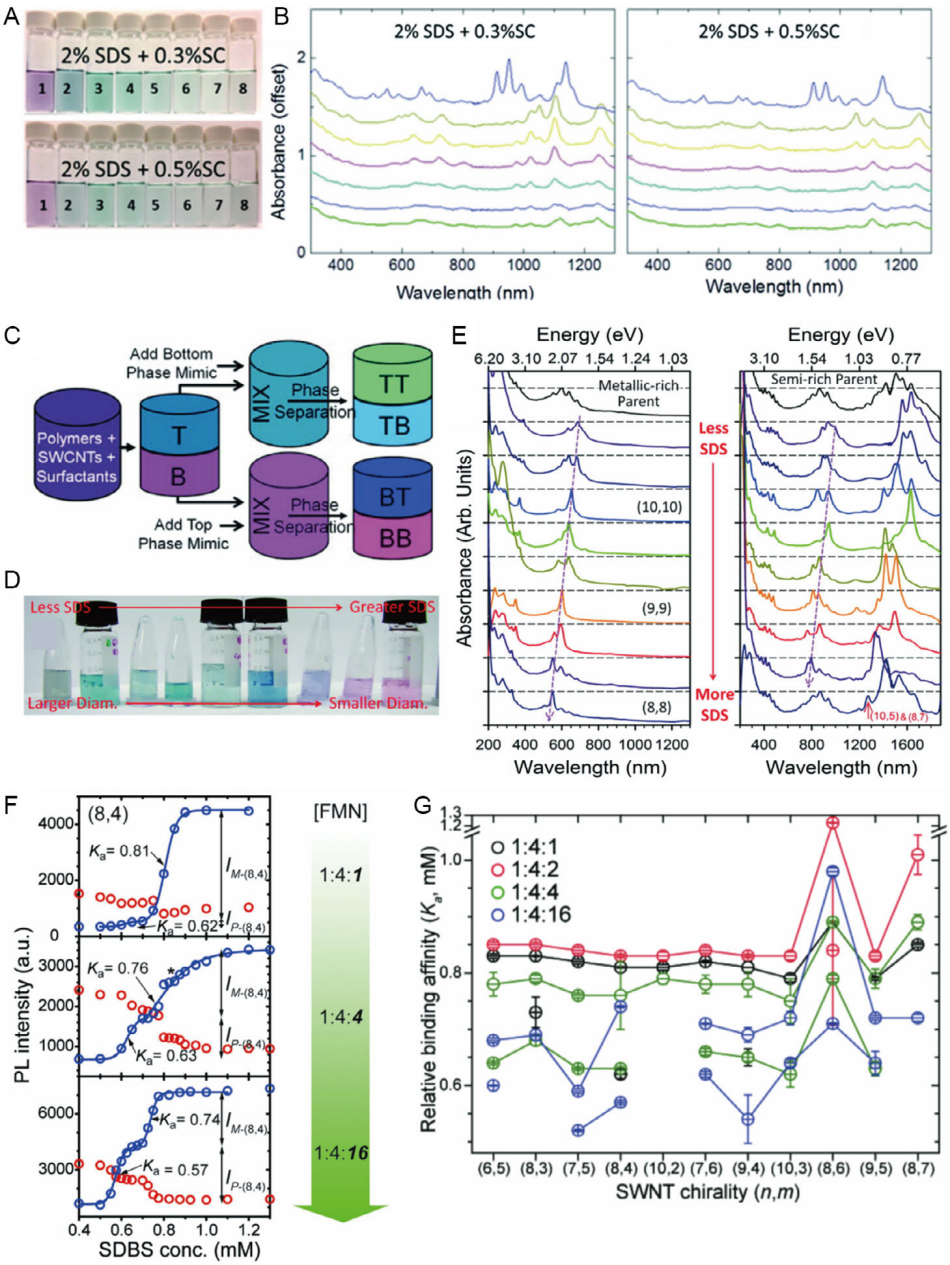
Dispersant concentration effect in GCC, ATPE, and optical titration. A) Photograph and B) absorption spectra of eluants in GCC created using mixed SDS/SC. Reproduced with permission.^[^ ^220^ ^]^ Copyright 2015, American Chemical Society. C) Schematic of the ATPE, multiple stage of separation performed by adding aliquots of the opposite phase with a different ratio of SDS/DOC. D) Photograph of SDS gradient ATPE‐separated *s*‐SWNT and E) absorption spectra of *m*‐ and *s*‐SWNT produced by *d*_t_ sorting by increasing the concentration of SDS. Reproduced under terms of the CC‐BY license.^[^ ^185^ ^]^ Copyright 2015, American Chemical Society. F) *K*_a_ changes according to initial FMN concentrations. SDBS‐ and FMN‐SWNT‐derived PL intensity traces. G) Overall trends of *K*_a_ by chirality and handedness. Reproduced with permission.^[^ ^210^ ^]^ Copyright 2017, American Chemical Society.

In 2015, Jain and co‐workers^[^ ^220^ ^]^ reported the effect of dispersant concentration on SWNT sorting using the GCC method with mixed SDS/SC. Photographs of eluents obtained by eluting a series of columns with fixed SDS concentrations (i.e., 2%) while increasing SC concentrations are given in **Figure** **22**A. The corresponding absorption spectra of the eluants (Figure 22B) show that the intensities of the absorbance maxima decrease as the SC concentration is increased, showing that a decrease in the numbers of SDS on the SWNT surface results in a reduced interaction with gel and consequent more rapid elution. This trend is dependent on *d*_t_ because of differences in the morphologies of the dispersant surrounding the SWNTs. These observations suggest the possibility of separating the desired SWNT types utilizing a controlled ratio of mixed dispersants.

In 2015, Fagan and co‐workers^[^ ^185^ ^]^ evaluated the effect of DOC/SDS ratios on SWNT sorting using the ATPE method. ATPE was performed using a continuous repetitive extraction process, as shown in Figure 22C. Increasing SDS concentration results in extraction of individualized *s*‐SWNTs with distinct colors (Figure 22D) and absorption spectra (Figure 22E). In 2017, Sim and co‐workers^[^ ^210^ ^]^ quantitatively determined how *K*_a_ varies according to increasing FMN concentrations utilizing SDBS titration of FMN‐wrapped SWNTs.^[^ ^210^ ^]^ As shown in Figure 22F, *K*_a_ is obtained from PL intensity changes of SWNT. The results show that *K*_a_ is dependent on FMN concentration and SWNT type (Figure 22G) and that it reaches a maximum value at a specific FMN concentration. Decreases of *K*_a_ at higher than the specific FMN concentration are suggested to result from torsional deformation of the SWNT caused by increased numbers of helically twisted FMN. In addition, increasing FMN concentration augments the difference in *K*_a_ of SWNT handedness with different vdW interaction.

#### 5.2.3 pH

**Figure 23:**
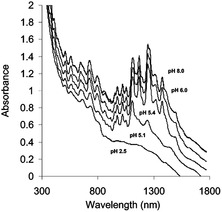
Optical transitions in absorption spectra are quenched by acidifying SWNTs dispersed by SDS. Reproduced with permission.^[^ ^221^ ^]^ Copyright 2003, American Chemical Society.

pH affects the surrounding ions and charge environment of both the SWNT and dispersant wrapping concomitantly. As a result, control of pH enables maximization of differences in *K*_a_ according to SWNT type and the sorting efficiency. Strano and co‐workers^[^ ^221^ ^]^ reported that protonation induces *d*_t_‐dependent optical bleaching (i.e., absorption intensity, fluorescent emission, and RRS intensity) of SWNTs coated by SDS in oxygenated acidic solutions. The protonation sensitivity is a consequence of bandgap differences in the SWNTs. *m*‐SWNTs are protonated near neutral pH, showing more reactivity in the acidic solution. In **Figure** **23**, absorption spectra show that the magnitude of *e* ^S^_11_ absorbance of SDS‐dispersed SWNTs decreases with increasing bandgap and decreasing pH. The results of Raman analysis show that *m*‐SWNT is more sensitive to pH changes than *s*‐SWNT. When interpreted from the point of view of redox chemistry, the pH effect can be explained as a *p*‐doping phenomenon using Equation (13).

(13) $$4\text{SWNT}+O_{2}+4H^{+}⇌4{\text{SWNT}}^{+}+2H_{2}O$$

where the Fermi level of pristine SWNT is about –4.7 eV, and the standard electrochemical potential of the oxygen/water redox couple ranges from –4.83 to –5.66 eV from pH 14 to at pH 0 according to the Nernst equation.^[^ ^222^ ^]^ The smaller *d*_t_ of *s*‐SWNT corresponds to a larger bandgap and, thus, *s*‐SWNT with larger large *d*_t_ values are more easily oxidized. Similarly, as the pH decreases, redox reactions of *m*‐SWNT, which has a continuum in the band structure, occur more readily than they do in *s*‐SWNTs.

**Figure 24:**
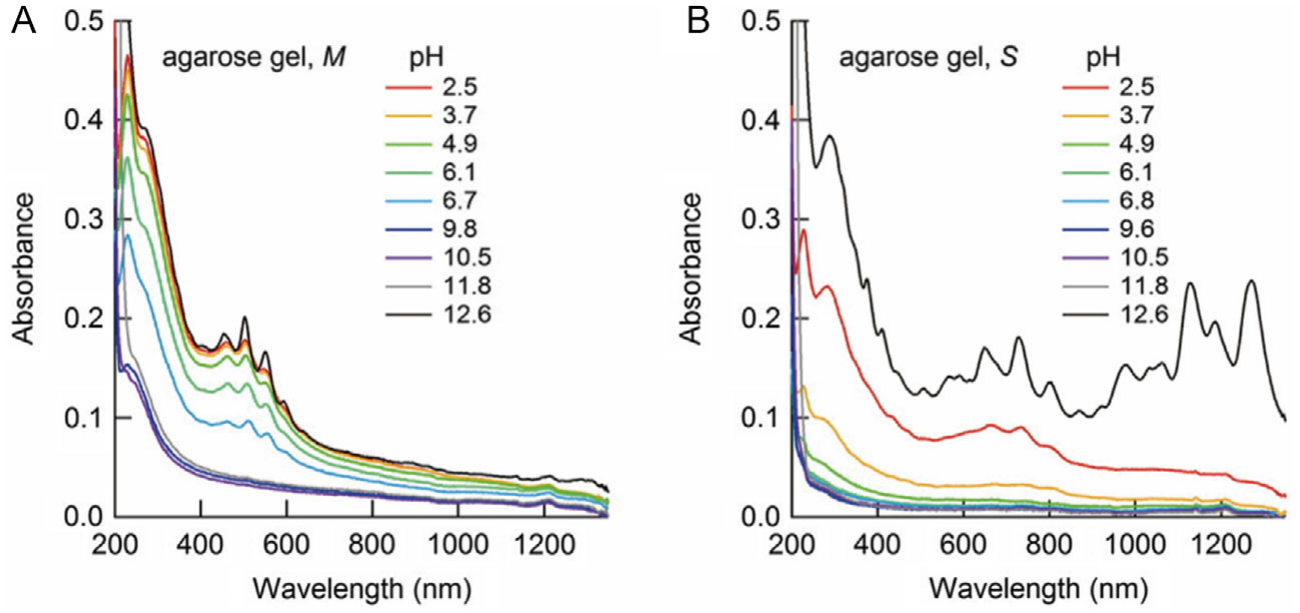
pH effect on sorting SWNTs using GCC (A‐B) pH‐dependent adsorption of the SWNTs onto the agarose gel. Absorption spectra of the fractions of A) *m*‐SWNT and B) *s*‐SWNT desorbed onto agarose gel. Reproduced with permission.^[^ ^223^ ^]^ Copyright 2013, American Chemical Society.

In 2013, Hirano and co‐workers^[^ ^223^ ^]^ reported that the interaction of *m*‐SWNT and *s*‐SWNT with a gel used in SDS promoted in GCC varies according to pH. **Figure** **24**A,B shows absorption spectra by dispersions obtained of *m*‐SWNT and *s*‐SWNT absorbed on gels as a function of pH. In each case, the absorption peaks increase as pH decreases, except at pH 12.5, and the increase of the peaks associated with *m*‐SWNT begins to increase at a higher pH than those in the spectrum of *s*‐SWNT. This difference stems from the presence of a continuum in the *m*‐SWNT band structure, so that charge transfer to become more *p*‐type of SWNT occurs more rapidly. In addition, SDS, an anionic dispersant, more tightly binds to *m*‐SWNT, which reduces interaction with the gel.

**Figure 25:**
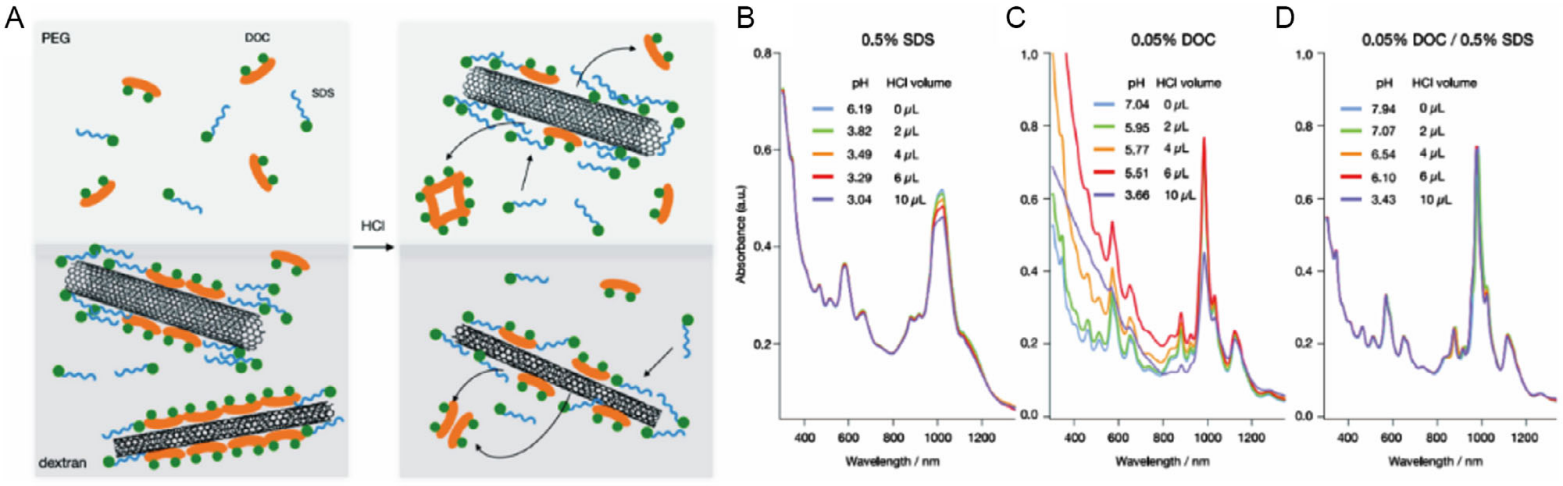
pH effect on sorting SWNTs by ATPE using a mixed SDS/DOC system. A) Schematics for pH‐driven ATPE sorting showing small and large *d*_t_ SWNTs wrapped in SDS/DOC before and after addition of HCl. B–D) Absorption spectra of SWNTs dispersed in water with B) 0.5% SDS, C) 0.05% DOC, and D) 0.5% SDS/0.05% DOC upon sequential addition of 0.5 m hydrochloric acid. Reproduced with permission.^[^ ^224^ ^]^ Copyright 2019, American Chemical Society.

Due to the difference in isoelectric points of ionic moieties in dispersants, changing the pH of a mixed dispersant system can be applied to sorting. Controlling sorting by varying dispersant concentrations has been used in ATPE to overcome some limitations. The reversible nature of redox coupling between a dispersant and SWNT can be used as a control element to regulate separation in ATPE that is difficult to perform only by controlling the concentration of a dispersant and ratio in mixed dispersants. In 2019, Li and co‐workers^[^ ^224^ ^]^ applied pH control to sorting desired *d*_t_ and electronic types of SWNTs using ATPE. **Figure** **25**A shows a schematic that shows the relationship for binding between SWNT and dispersant as a function of acid concentration in a mixed DOC/SDS system. The SWNT species dependence of the degree of transition from the bottom phase to the top phase is maximized using the mixed dispersant system. Figure 25B–D shows absorption spectra obtained by adjusting pH after SWNT dispersion using SDS, DOC, and DOC/SDS mixed dispersant. Inspecting these spectra shows that in the case of SDS, no significant change in absorbance takes place even when the pH decreases, but in the case of DOC, the absorbance peak decreases as pH is lowered. This result is due to protonation of the carboxylate group in DOC that has a high p*K*_a_ value of 6.58. In comparison, the p*K*_a_ of the sulfonic acid group in SDS is about 1.9 and, therefore, charge of SDS does not change in the pH range explored. However, as shown in Figure 25D, when a DOC and SDS mixture is used, no significant change occurs in the optical transitions when pH is changed. The reason behind for this is that when DOC is aggregated, SDS binds to defective areas on the SWNT surface to maintain the dispersed state. As the pH decreases in the DOC/SDS mixed system, SDS binds to aggregated positions in tightly DOC‐wrapped SWNTs, making them hydrophobic. Thus, transition from the hydrophilic DEX bottom phase to the top hydrophobic PEG phase occurs.

#### 5.2.4 Aging Time

To individualize bundled SWNTs with a dispersant, a dispersion is created by applying energy such as sonication. After dispersion, the system adjusts in a time‐dependent manner to form a thermodynamically stable equilibrium. In this process, rebundling/unbundling of SWNT in the dispersion competes with interactions between the SWNT and dispersant. Moreover, the dispersant itself rearranges to form thermodynamically more stable forms. The timing for the occurrence of these changes is dependent on the nature of the dispersant.

**Figure 26:**
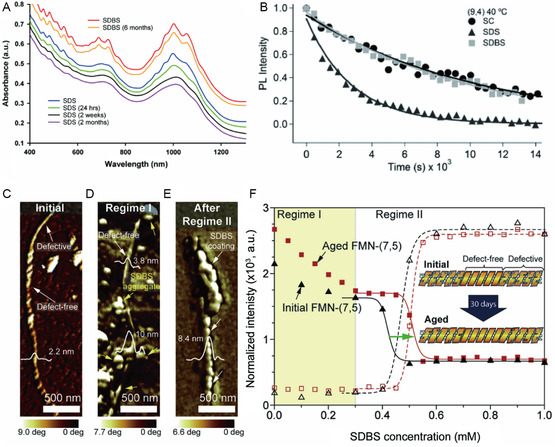
Aging effect on binding affinity between SWNT and dispersants. A) Absorption spectra change with time associated with different dispersants. Reproduced with permission.^[^ ^219^ ^]^ Copyright 2010, American Chemical Society. B) PL intensity change with time associated with different dispersants at 40 °C. Reproduced with permission.^[^ ^56^ ^]^ Copyright 2006, American Chemical Society. C–E) AFM phase images of samples at the C) initial, D) regime I, and E) after regime II stages. (F) Normalized PL intensity trajectories generated by SDBS titration showing *K*_a_ of FMN‐(7,5) SWNTs at initial and aged stages. Reproduced with permission.^[^ ^76^ ^]^ Copyright 2018, Elsevier.

In 2010, Blanch and co‐workers^[^ ^219^ ^]^ reported that the degree of dispersion varies with time in a manner that is dependent on the dispersant and the SWNT growth method. When dispersions are prepared using SDS and larger *d*_t_ SWNTs (i.e., >1 nm) generated using the arc discharge method, clearly distinct absorption peaks become featureless within 24 h and continue to broaden over time (**Figure** **26**A). In comparison, when HiPco SWNTs were dispersed in SDS, no significant change in absorbance occurs. The *d*_t_ range for HiPco SWNTs is 0.8–1.0 nm, and 1.3–1.7 nm for arc‐grown SWNTs.^[^ ^225^ ^]^ As *d*_t_ of SWNTs increases, the binding energy between the SWNTs increases, so that bundling occurs more easily.^[^ ^226^ ^]^ Therefore, SDS associated with larger *d*_t_ SWNTs is prone to reaggregation. However, in the case of SDBS, which is in the same linear dispersant as SDS, no significant change takes place. This is a consequence of the fact that the dispersion ability of SDBS results from *π*–*π* interactions of its phenyl ring with SWNTs.

Similarly, McDonald and co‐workers^[^ ^56^ ^]^ reported in 2006 that the rate of PL quenching caused by rebundling of SWNT at 40 °C is higher in SDS compared to that in SC and SDBS (Figure 26B).^[^ ^56^ ^]^ The dispersant‐related changes with time are caused by differences in the properties of SDS and SDBS, the latter containing an aromatic ring, which affects binding affinities.

In 2018, Park and co‐workers^[^ ^76^ ^]^ reported that defective parts of SWNTs that are not completely wrapped by FMN are initially replaced by the co‐titrant SDBS, and that this phenomenon is involved in the time‐dependent changes that occur. Figure 26C contains an atomic fore microscopy (AFM) image at the initial point after SWNT is wrapped with FMN. The image shows that a defective FMN assembly exists at the end of the FMN‐SWNT chain. In the process of titration of the sample (regime I, Figure 26D), SDBS becomes aggregated to SWNT at the site of defective FMN assembly. Upon increasing SDBS concentration, SDBS becomes fully wrapped about the SWNT in the form of a micellar structure (regime II). Photoluminescence (PL) intensity changes in Figure 26F show that *K*_a_ increases after the initial sample is aged for 30 d, demonstrating that the dispersant‐wrapped assembly stabilizes over time. The results suggest that the variation of binding affinity over time might depend on the type of dispersant.

### 5.3 Scaling of Binding Affinity Change According to Intrinsic and Extrinsic Parameters

**Figure 27:**
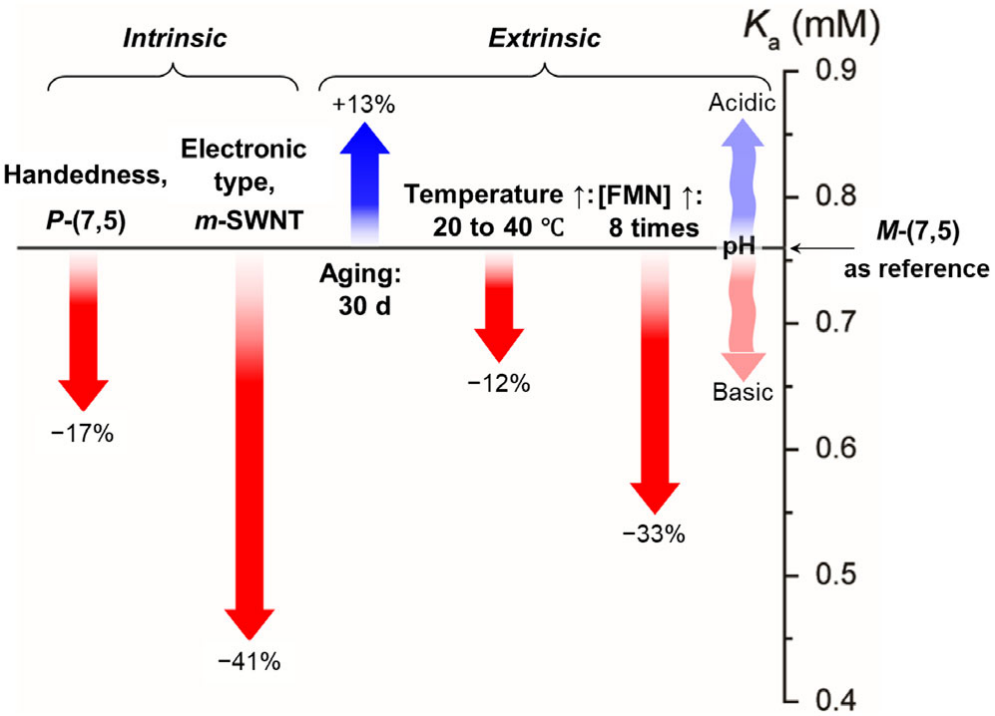
Comparative plot of the change in *K*_a_ values due to intrinsic (i.e., handedness and electronic type) and extrinsic (i.e., aging time, temperature, dispersant concentration and pH) parameters based on the *K*_a_ value that appears during SDBS titration of FMN/*M*‐(7,5)‐SWNT. pH effect is based on the other dispersant system.

The effects of intrinsic and extrinsic parameters (i.e., handedness, electronic type, aging time, temperature, dispersant concentration and pH) on *K*_a_ of dispersants can be assigned relative values. The plot in **Figure** **27** compares *K*_a_ in terms of these parameters, utilizing the *K*_a_ value (i.e., 0.76 mM)^[^ ^210^ ^]^ between FMN and *M*‐(7,5) SWNT. Chirality was not included in this consideration owing to larger variations according to SWNT.

Intrinsic parameters associated with SWNT structures that contribute to *K*_a_ values of dispersants are displayed in the left panel of Figure 27). First, among the intrinsic and extrinsic parameters, the electronic type of SWNT brings about the greatest differences in *K*_a_. Compared to (7,5) s‐SWNT, the *m*‐SWNT ensemble which has a similar *d*_t_ has a 41% lower *K*_a_ value.^[^ ^75^ ^]^ This large *K*_a_ difference enables flavin to selectively generate *s*‐SWNT with high purity.^[^ ^153^ ^]^ In addition, flavin on the SWNT surface forms a stable *p*‐type helix,^[^ ^155^ ^]^ and different interactions resulting from this wrapping modes lead to a reduced the *K*_a_ for *P‐*SWNT by 17% relative to that of *M‐*SWNT.^[^ ^210^ ^]^

Extrinsic parameters associated with external environmental factors also control *K*_a_ (right panel of Figure 27). The flavin assembly on the SWNT surface forms a thermodynamically more stable wrapping structure as time increases, in conjunction with a 13% increase in the *K*_a_.^[^ ^76^ ^]^ On the other hand, increasing temperature during dispersant titration decreased *K*_a_ due to its effect on intermolecular interactions of FMN.^[^ ^75^ ^]^ Also, FMN concentration, other than the optimum, also effects *K*_a_ and an increase in the FMN concentration results in a 33% decreased *K*_a_.^[^ ^210^ ^]^ Finally, as observed in other dispersant systems,^[^ ^223^ ^]^ decreases in pH result in *p*‐doping of SWNT causing improved interactions with the anionic phosphate group in FMN. Unlike for intrinsic parameters of SWNT, control of extrinsic parameters can lead to improved SWNT separation efficiencies by maximizing the *K*_a_ differences.

## 6 Conclusion

SWNTs are considered promising next‐generation nanomaterials for a wide range of applications due to their exceptional optoelectronic and mechanical properties. However, SWNTs produced via batch production are inevitably a mixture of types, including different electronic types, chiralities, and handedness. To fully utilize their properties, it is crucial to isolate high‐purity SWNTs with defined chirality. For example, *s*‐SWNTs with purities over 99.9999% are ideal for high‐end field‐effect transistors with channel widths below 10 nm.^[^ ^3^, ^5^ ^]^ Achieving such high‐purity criteria requires more selective SWNT sorting schemes.

Various dispersant‐based systems have been developed to sort SWNTs according to their electronic type, chirality, and handedness. The minute differences in the *K*_a_ of dispersants to SWNTs with different chiralities have enabled the differentiation of dispersant‐SWNT assemblies using a range of techniques and reagents. The changes in *K*_a_ resulting from these factors are associated with the stability of the dispersant supramolecular structure. Therefore, as introduced in this review, a comparison of intrinsic and extrinsic parameters affecting *K*_a_ can suggest future directions for SWNT sorting. Future research should not only focus on the numerical value of binding affinity but also on narrowing its width. This is essential because overlaps in the binding affinity width currently lead to reduced purity in the purified SWNT. Addressing this issue by refining and sharpening the binding affinity will be a crucial topic in forthcoming studies. It is essential to understand not only the thermodynamic approach using the aforementioned binding affinity, but also the kinetic approach^[^ ^227^ ^]^ for binding control. A comprehensive understanding of these parameters is essential for separating single SWNT species and designing tailored dispersants for SWNT sorting. In addition, recycling of dispersants^[^ ^195^ ^]^ and economical methods that use less expensive equipment are expected to be advantageous for separating carbon nanotubes in the future. Various schemes developed for SWNT sorting have also been applied to other 1D and 2D materials, including graphene, hexagonal boron nitride, and transition metal dichalcogenides.^[^ ^228^ ^]^ Additionally, precise assembly of dispersants on SWNTs based on chirality can be used to functionalize SWNTs with various entities at specific locations.^[^ ^229^, ^230^ ^]^ These controlled defects can be utilized as stable single‐photon sources for quantum computing systems.^[^ ^231^ ^]^

## Conflict of Interest

The authors declare no conflict of interest.
